# CEACAM1 promotes melanoma metastasis and is involved in the regulation of the EMT associated gene network in melanoma cells

**DOI:** 10.1038/s41598-018-30338-4

**Published:** 2018-08-08

**Authors:** Daniel Wicklein, Benjamin Otto, Anna Suling, Eva Elies, Georg Lüers, Tobias Lange, Susanne Feldhaus, Hanna Maar, Jennifer Schröder-Schwarz, Georg Brunner, Christoph Wagener, Udo Schumacher

**Affiliations:** 1 Institute of Anatomy and Experimental Morphology, University Cancer Center, University Medical-Center Hamburg-Eppendorf, Hamburg, Germany; 2 Eppendorf AG, Hamburg, Germany; 3 Department of Medicine I, University Medical Center Hamburg-Eppendorf, Hamburg, Germany; 4 Department of Medical Biometry and Epidemiology, University Medical Center Hamburg-Eppendorf, Hamburg, Germany; 5 Department of Cancer Research, Fachklinik Hornheide, Münster, Germany; 6 Center for Diagnostics, University Medical Center Hamburg-Eppendorf, Hamburg, Germany; 7 Present Address: NeraCare GmbH, Bönen, Germany

## Abstract

We investigated the functional role of CEACAM1 in a spontaneous metastasis xenograft model of human melanoma in scid mice using BRAF wildtype MeWo cells with and without RNAi mediated knockdown of CEACAM1. Tumors from the xenograft model were subjected to whole genome expression analysis and metastasis was quantified histologically. Results and identified markers were verified using tissue samples of over 100 melanoma patients. Knockdown of CEACAM1 prolonged the animals’ survival by significantly reducing subcutaneous growth of MeWo tumors and spontaneous lung metastasis. Microarray analysis revealed a strong influence of CEACAM1 knockdown on the network of EMT associated genes in the xenograft tumors (e.g. downregulation of BRAF, FOSL1, NRAS and TWIST). IGFBP7 and Latexin (highest up- and downregulated expression in microarray analysis) were found to be associated with longer and shorter survival, respectively, of melanoma patients. High FOSL1 and altered TWIST1 expression were found to be correlated with shortened survival in the cohort of melanoma patients. After a stepwise selection procedure combining above markers, multivariate analysis revealed IGFBP7, Latexin and altered TWIST to be prognostic markers for death. CEACAM1 could be a target for melanoma therapy as an alternative to (or in combination with) immune checkpoint and BRAF inhibitors.

## Introduction

Malignant melanoma is an extremely dangerous disease with high mortality rates due to the aggressive metastatic potential of melanoma cells. Although the development of new therapies for patients with already metastasized melanoma over the last few years resulted in prolonged survival, for a considerable number of patients these new therapies still do not achieve stable remission for more than a few months (see^1^ for current review). For example, treatment with agents directed against mutated BRAF alone eliminates visible metastases shortly after first administration; however, due to resistance development, metastatic disease reoccurs after 6–8 months^2,3^. Additionally, BRAF therapy is contraindicated for patients with BRAF wildtype melanoma und thus this treatment is not feasible for roughly half of the patients. Current ideas for better disease control include a combination of treatments. In cases with BRAF mutated melanoma, combining BRAF and MEK inhibitors further delays the development of resistance to about 11 months and patients with metastases at fewer than 3 organ sites and low LDH can even be stabilized for years^2^. Finding additional therapeutic targets on melanoma cells, preferably molecules, which play a functional role in metastasis, could greatly enhance chances for developing such combination therapies.

CEACAM1 (also known as C-CAM, biliary glycoprotein (BGP) and CD66a) belongs to the CEA protein family and as such to the immunoglobulin superfamily. It is a complex glycoprotein located in the outer membrane of cells (see^4^ for an extensive review). CEACAM1 expression was found to be reduced in the early phases of some cancers (e.g. colon^5^ and breast^6^) and was thus judged as a tumor suppressor gene in these tumors^7^. In some types of cancer (e.g. bladder^8^, gastric^9^ and pancreatic^10^) however, high expression of the molecule was associated with reduced overall survival or metastatic spread. One of the latter types is melanoma where a high expression of CEACAM1 was found to be correlated with metastasis^11–13^ and CEACAM1 on melanoma cells was shown to be protective against T cell attack^14^. Furthermore, CEACAM1 can interact with integrins^15^ and also enhances invasion and migration of melanoma cells *in vitro*^16^. Taking these findings together, we hypothesized that CEACAM1 might have direct functional relevance for melanoma metastasis involving melanoma cell-intrinsic mechanisms which are independent of T cell activation. We chose to investigate this hypothesis using a spontaneous metastasis xenograft model of human BRAF-wildtype melanoma in scid mice (lacking functional T and B cells), modeling the whole metastatic cascade.

## Material and Methods

### Cell culture

MeWo cells were obtained and cultivated as previously described^11^. The cell line was certified by the DSMZ (Leipniz Institute DSMAZ-German Collection of Microorganisms and Cell Cultures) Cell culture medium for selection and cultivation of transfected cells was additionally supplemented with 0.5 µg/ml puromycin (Life Technologies).

### RNAi mediated knockdown of CEACAM1

CEACAM1 knockdown in MeWo cells (MeWo CEACAM1 kd) was achieved by an shRNA mediated approach: A 65 bp DNA oligomer containing a 19 bp anti-CEACAM sequence (CTCACAGCCTCACTTCTAA) was inserted into the pSIREN RetroQ vector (Clontech, Saint-Germain-en-Laye, France) according to the manufacturer’s instructions. The anti-CEACAM sequence chosen was directed against the N-terminal domain shared by all CEACAM-family members to avoid upregulation of other members as a possible compensation for the lack of CEACAM1. The sequence was checked for potential off-target effects using PubMed BLAST. The same vector containing a sequence against firefly luciferase was used to generate a transfected control cell line (MeWo Luc). MeWo cells were transfected non-virally and transfected cells selected as described^10,17^. Briefly, cells were transfected using FuGENE6 (Roche, Mannheim, Germany) and 3 cells per well of a 96-well plate were deposited in the limiting dilution after puromycin selection. MeWo CEACAM1 kd sublines were screened for CEACAM1 expression using flow cytometry.

### Proliferation and migration/invasion *in vitro* assays

The Cell Proliferation Kit II (XTT, Roche Diagnostics, Mannheim, Germany) was used for *in vitro* proliferation assays according to the manufacturer’s instructions with 5 × 10^4^ cells seeded per well and 48 h proliferation time. The experiment was repeated twice (one week interval each) with 6 replicates (wells) each time (total of n = 18 each group).

Invasive potential was tested *in vitro* using the 8 µm, 24-multiwell BioCoat System (BD) with Calcein AM (eBioscience,), according to the manufacturer’s instructions. 1 × 10^5^ MeWo CEACAM1 kd or MeWo Luc cells were seeded per well. Fluorescence signals were analyzed with a Genios Reader (Tecan, Männerdorf, Switzerland). The experiment was repeated once after one week with 3 replicates (wells) each time (total of n = 6 for each group).

### Flow Cytometry

Flow cytometry for CEACAM1, 3, 5, 6, 8 and 21 was performed as described^10,17^. Briefly, melanoma cells were stained with mouse anti-human PE-labeled CEACAM1/CD66a (R&D, Wiesbaden, Germany), PE-labeled anti-CEACAM3 (Sino Biological, Beijing P.R. China), FITC-labeled anti-CEACAM5 (Bio-Rad/AbD Serotec, Munich, Germany), APC-labeled anti-CEACAM6 (R&D, Wiesbaden, Germany), FITC-labeled anti-CEACAM8 (Miltenyi, Bergisch-Gladbach, Germany), FITC-labeled anti-CEACAM21 (antibodies online.com, Aachen, Germany) or the corresponding isotype controls (Miltenyi). For CEACAM3,5,6,8,21 cells were also fixed (4%PFA) and permeabilized (1% Saponin) prior to staining. IGFBP7 was demonstrated by incubation with anti-human IGFBP7 (ab89347, Abcam, Cambridge, United Kingdom) or corresponding mouse IgG1 isotype control (Dako, Hamburg, Germany) followed by staining with allophycocyanin-labeled goat anti-mouse Ig (BD, Heidelberg, Germany). Intracellular Latexin was shown by serial incubations of paraformaldehyde fixated cells with goat anti-Latexin (AF3246, R&D) or corresponding goat isotype control (Dako) followed by biotinylated rabbit anti-goat (Dako) and allophycocyanin-labeled streptavidin (BD). Stained cells were subjected to fluorescence assisted flow cytometry on a FACSCalibur (BD). Files were analyzed using Win MDI 2.9 software.

### Xenograft mouse model of human melanoma

The experiment was carried out as previously described^11^. Briefly, 12 scid mice in each group received subcutaneous injections of 10^6^ MeWo cells with CEACAM1 knockdown (CEACAM1 kd) or MeWo Luc as controls, respectively. Mice with ulcerated tumors or tumors with an estimated mass exceeding 10% of the respective animal’s weight were euthanized immediately.

The methodology for carrying out the animal experiments was consistent with the UKCCR guidelines for the welfare of animals in experimental neoplasia^18^. The experiment was recommended and supervised by the institutional animal welfare officer and approved by the local licensing authority (Behörde für Soziales, Familie, Gesundheit, Verbraucherschutz; Amt für Gesundheit und Verbraucherschutz; Billstr. 80, D-20539 Hamburg, Germany). All methods were performed in accordance with the relevant guidelines and regulations by the local authorities.

All animals used were pathogen-free Balb/c severe combined immunodeficient (scid) aged 9–14 weeks with a weight of 25–30 g at the beginning of the experiments. The mice were housed in filter-top cages, provided food and water ad libitum and their condition was monitored daily. Apart from visible tumors, the general condition of the animals was evaluated by a standardized in house scoring system based on movement/behavior, weight development, food and water intake and fur condition. The mice were killed by cervical dislocation after having been anesthetized by intraperitoneal injection of a weight-adapted dose (10 µl/g bodyweight) of a mixture of 1.2 ml Ketamin (Gräub AG, Bern, Switzerland), 0.8 ml Rompun (Bayer AG, Leverkusen, Germany) and 8 ml saline.

### Immunohistochemistry

Immunohistochemistry using sections of paraffin-embedded tissues was carried out as previously described^11,19^. Antibodies used for IGFBP7 and Latexin staining were mouse anti-human IGFBP7 (Abcam) and goat anti-Latexin (R&D) as described for flow cytometry. For IGFBP7 staining, samples were incubated in a steamer for 20 min in S1699 (Dako) prior to incubation with antibody.

Stained slides were scanned by a Mirax microscope (Zeiss, Jena, Germany) and the Panoramic Viewer software (3D Histech, Budapest, Hungary) was used to take images.

### Quantification of lung metastasis

Lung metastases were quantified as previously described^20^. Briefly, whole lungs were sectioned into 1 mm thick slices and embedded first in agar and afterwards in paraffin wax. The embedded lung tissue was serially sectioned at 5 µm thickness and every tenth section from the middle of the tissue block was HE stained. The number of metastases in 10 sections was counted and total lung metastases were calculated for each animal based on the respective total number of slices minus a correction factor.

### Nucleic acid extraction and real-time PCR

DNA and RNA extraction from murine blood and tumors, quantification of human melanoma cells by real-time polymerase chain reaction (PCR) and quantitative real-time PCR (qRT-PCR) for IGFBP7 and Latexin expression (with GAPDH as housekeeping control) were performed as previously described^19^. Each experiment was performed twice with 5 replicates each time. Primers used for human IGFBP7 and Latexin quantification were:

ACCGCACCCCGCCATGGAG (IGFBP7 forward);TCTGGAGGTTTATAGCTCGGCACCT (IGFBP7 reverse);TTTTCCGCCTCGGCAACCGG (Latexin forward) andTTGGCCTCATAAGCTAGATGCCAGT (Latexin reverse).

### Microarray protocol

Total RNA was extracted using Trizol Reagent (Invitrogen) followed by DNA digestion (TURBO DNA-free; Ambion) according to the manufacturer’s instructions. Total RNA was cleaned up using RNeasy mini kit according to the manufacturer’s instructions (Qiagen). Labeling, hybridization on the Affymetrix microarray chips (Human Whole Genome U133 Plus 2.0) and image data processing were completed according to the Affymetrix 3′ IVT standard protocol using the GeneChip Fluidics Station 450, Affymetrix 3 G scanner and command console software.

For data processing an alternative cdf-file (HGU133Plus2_Hs_ENSG_14.0.0) obtained from the Brainarray database^21^ was used. Signals were adjusted using RMA background correction procedure^22^ and quantile normalization^23^ (Bioconductor package simpleaffy version 1.16.0) on the R statistical platform (version 2.6.2).

Significantly regulated genes were determined using an unequal variance based, unpaired T-test followed by bootstrapping procedure to correct for multiple testing. As cut-off criteria a raw p-value of 0.05, a corrected p-value of 0.05 and a signal-log-ratio (SLR) of ±0.6 were set as thresholds.

### Subjects and Tissue Specimens

Following written informed consent of the patients, tissue samples were recruited for this study. Tissue collection was approved by the local institutional review board of the Westphalian Wilhelms University in Münster, Germany. All research was performed in accordance with relevant guidelines/regulations. Melanomas across AJCC 2009 stages IA-IIIC were chronologically recruited between 1983 and 2006 at the Skin Cancer Center Hornheide in Münster. Melanomas were reviewed, following hematoxylin-eosin staining, to confirm diagnosis and verify ≥50% of Breslow thickness.

### Statistical analysis

Continuous variables are displayed using boxplots and are additionally described using mean, standard deviation, median, minimum and maximum.

The influence of the markers CEACAM1, Latexin, IGFBP7, TWIST and FOSL1 (Fra-1) on patient survival was analyzed in a first step using Log-rank tests and separate age-adjusted Cox regression models. Results are visualized in Kaplan-Meier survival curves together with 95%-confidence intervals (CI) and displaying number of patients at risk for some time points. Kaplan-Meier curves are halted at ten years, as afterwards the estimation uncertainty increases due to a strongly decreasing number of patients at risk^24^. Results of Cox regression are shown using hazard ratios (HR) together with 95%-CI. In a second step an age-adjusted multivariate Cox regression was calculated using all markers as predictors, which were backwards eliminated based on p-values (p < 0.05). To determine association between examined markers we used the phi-coefficient (ϕ) together with a chi-squared test.

Difference between CEACAM1 kd and Luc in *in vitro* outcomes proliferation and migration were analyzed using unpaired t-tests. As Latexin and IGFBP7 were collected at two points in time, these outcomes were analyzed with a linear regression model including the interaction of time and group, which was excluded if not significant. Results are presented using estimated group differences together with 95%-CI.

Outcomes of the xenograft model were survival, tumor weight, metastases, and number of circulating tumor cells (CTCs; at the time of death in the animals’ blood, determined by qRT-PCR). Survival of mice was defined as time until mice were euthanized when the tumor ulcerated or reached an estimated mass exceeding 10% of the respective animal’s weight. As no censoring was existent it was analyzed using a linear model which was moreover adjusted for the type of death (natural vs. euthanized). Tumor weight was analyzed in an analogous model. Number of metastases and CTCs were logarithmized and analyzed using a linear model, which was adjusted for survival time and tumor weight. Parameter estimates are presented together with 95%-CI.

A two-tailed p < 0.05 was considered to be statistically significant. All analyses were conducted using Stata 14.2 (StataCorp LP, College Station, Texas, USA).

## Results

### Knockdown of CEACAM1

After transfection of the human MeWo melanoma cells, puromycin selection and limiting dilutions, the new sublines were analyzed for CEACAM1, 3, 5, 6, 8 and 21 expression by flow cytometry.

**Figure 1:**
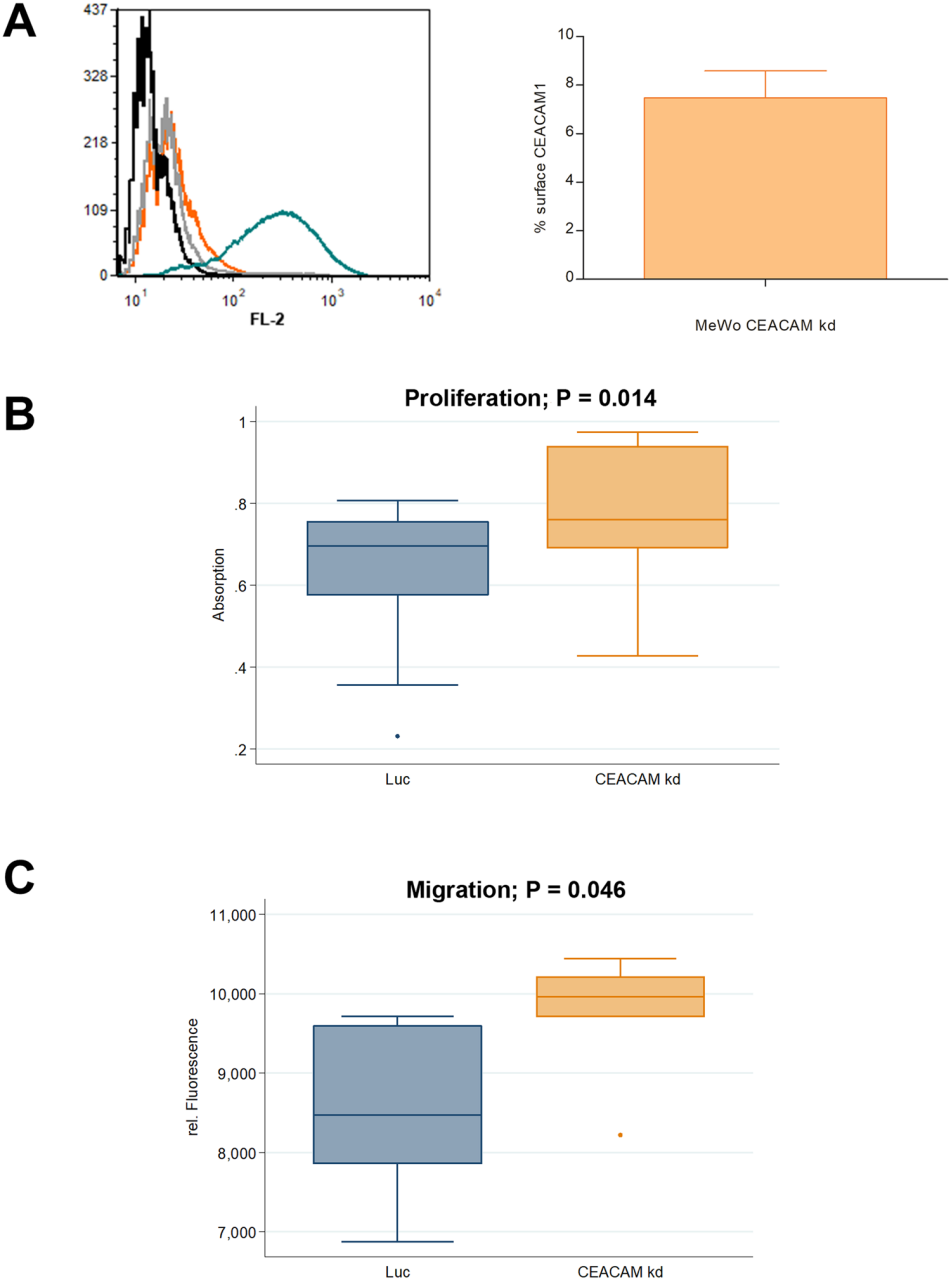
CEACAM1 knockdown in human melanoma cells and *in vitro* characterization. (**A**) Left Panel: Flow Cytometry, Histogram. Staining of MeWo CEACAM1 kd (orange curve, respective isotype control in grey) and MeWo Luc (teal, respective isotype control in black) for CEACAM1. Right Panel: In contrast to MeWo Luc, surface CEACAM1 is reduced by over 90% in MeWo CEACAM1 kd, given is the percentage of surface CEACAM1 on MeWo CEACAM kd cells as determined by flow cytometry (Staining and measurement repeated twice; respective MeWo Luc signals set as 100%). (**B**) *In vitro* XTT proliferation assay with MeWo CEACAM1 kd and MeWo Luc. A slight, but significant increase (p = 0.014) in proliferation was observed for MeWo CEACAM1 kd compared with the MeWo Luc controls. The experiment was repeated twice with 6 replicates (wells) each time (n = 18 each group). (**C**) *In vitro* migration in a transwell assay with MeWo Luc and MeWo CEACAM1 kd: Significant difference in migration between the two cell lines was observed (p = 0.046). The experiment was repeated once with 3 replicates (wells) each time (n = 6 for each group).

Five sublines with more than 90% reduced CEACAM1 expression were pooled and used for all further experiments (designated MeWo CEACAM1 kd). MeWo Luc cells with unchanged CEACAM1 expression but with puromycin resistance were used as controls (Fig. 1A). MeWo cells did not display any CEACAM3, 5, 6, 8 and 21 expression (Supplementary Fig. 1).

### *In vitro* proliferation and migration of MeWo cells increases after knockdown of CEACAM1

In an XTT assay, MeWo CEACAM 1 kd cells showed a significant increase in *in vitro* proliferation (+0.13, 95%-CI [0.03;0.24]; p = 0.014) compared with the MeWo Luc controls with unchanged surface CEACAM1 (Fig. 1B). Additionally, a significant increase in the cells’ ability for *in vitro* migration in a transwell assay was observed (+1253.0, 95%-CI [26.5;2479.5]; p = 0.046; Fig. 1C).

### CEACAM1 knockdown increases survival in a xenograft model of human melanoma

**Figure 2:**
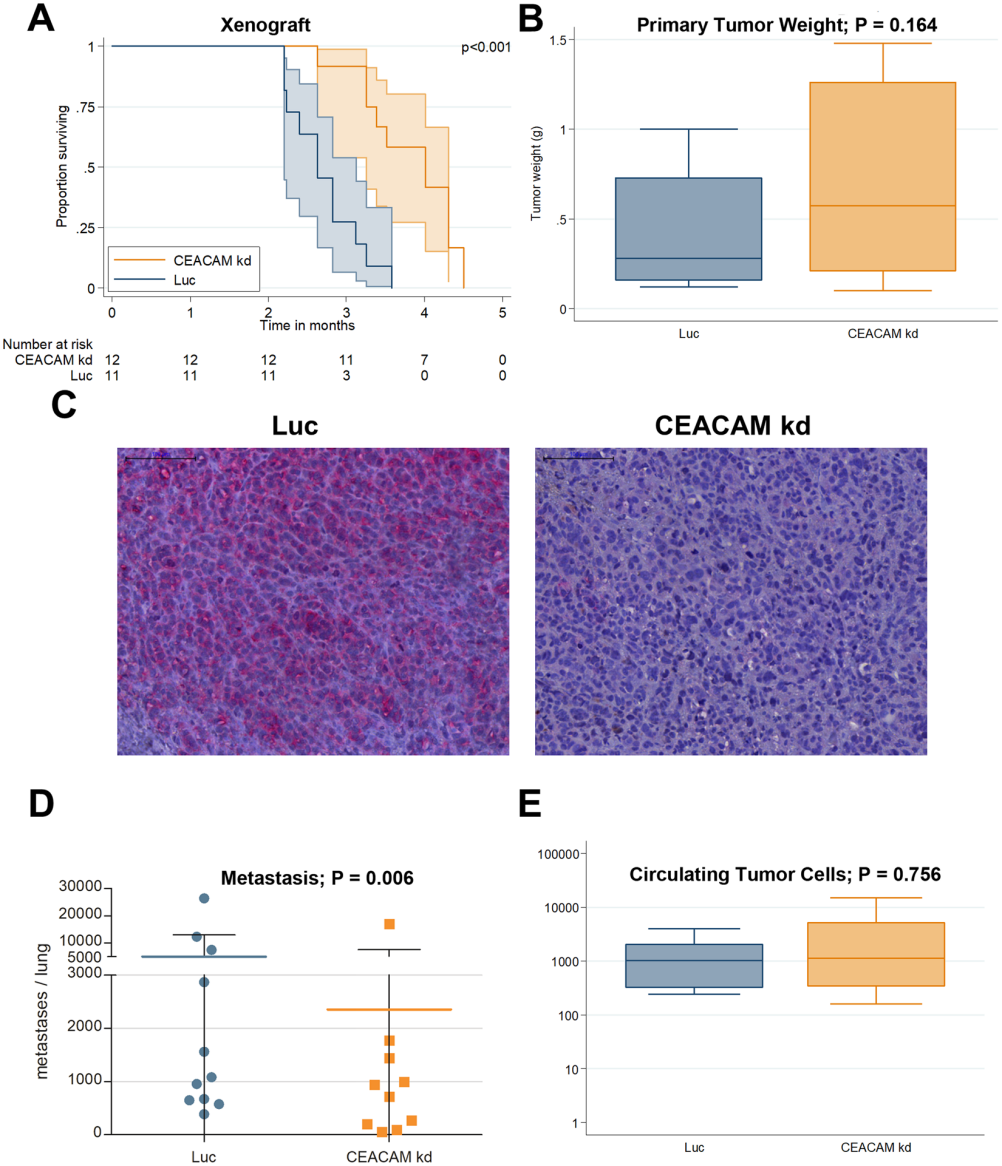
Spontaneous metastasis xenograft model of human melanoma. (**A**) Kaplan-Meier overall-survival analysis (OS) for scid mice with subcutaneously injected MeWo cells revealed significantly prolonged OS in mice inoculated with MeWo CEACAM1 kd 1 against MeWo Luc inoculated animals (p < 0.001). (**B**) Tumor weight at the time of death was not significantly different between the two groups (p = 0.164; n = 11 Luc group and n = 12 CEACAM kd group). (**C**) Immunohistochemical staining of paraffin embedded tumor tissue (xenograft MeWo tumors in scid mice) for CEACAM1 demonstrated the stability of the CEACAM1 knockdown in the subcutaneous tumors: High CEACAM1 was detected in the MeWo Luc tumors (left panel) whereas little to no CEACAM1 (in red) could be shown in the MeWo CEACAM1 kd tumors (right panel). scale bar: 100 µm. (**D**) Knockdown of CEACAM1 significantly decreases metastasis (adj. for survival time and tumor weight; p = 0.016; n = 11 Luc group and n = 10 CEACAM kd group). (**E**) CEACAM1 knockdown does not significantly alter the number of circulating tumor cells in the blood of scid mice (p = 0.756, n = 10 each group). Quantification of circulating (human) tumor cells in the blood of scid mice inoculated with MeWo CEACAM1 kd (orange) compared with controls (MeWo Luc, teal) by quantitative real-time PCR.

MeWo CEACAM1 knockdown (CEACAM1 kd) and MeWo Luc cells with unaltered CEACAM1 expression were injected subcutaneously into scid mice (n = 12 each group). One mouse from the Luc group died two days after injection and was therefore excluded from further analyses. All remaining animals developed subcutaneous tumors at the injection site and were euthanized when the tumor ulcerated. The tumors in the CEACAM1 kd group ulcerated later than the corresponding Luc tumors: Overall survival (OS; time from inoculation to euthanization) was significantly longer (+31.1 d, 95%-CI [18.2;44.0]; p < 0.001) in the CEACAM1 kd group (range 80 to 137, mean 116.6 ± 18.5 d, median OS 122 d, n = 12) compared with the Luc controls (range 67 to 109 d, mean 82.7 ± 14.0 d, median OS 80 d, n = 11; Fig. 2A). Tumor weight at the time of death was not significantly altered between the two groups (+0.13, 95%-CI [−0.06:0.33]; p = 0.164; Fig. 2B).

Immunohistochemical staining of paraffin embedded tumor tissue demonstrated the stability of the CEACAM1 knockdown in the subcutaneous tumors: Little to no CEACAM1 protein could be shown in the MeWo CEACAM1 kd tumors whereas high CEACAM1 was detected in the MeWo Luc tumors (Fig. 2C).

### CEACAM1 knockdown decreases metastasis in the xenograft model

Metastatic spread of the human melanoma cells was exemplary studied in the animals’ lungs using a histological approach: Metastases were counted in formalin-fixed and paraffin-embedded lung tissue after H.E. staining. Two lungs of the CEACAM1 knockdown group were lost due to an error during the fixation process, thus 10 lungs and 11 lungs were included in the analysis in the MeWo CEACAM1 kd and MeWo Luc groups, respectively.

Metastatic load (adjusted for survival time and tumor weight) in the Luc control group was 14.7 times higher (95%-CI [2.4;91.3]; p = 0.006) in comparison with the CEACAM1 kd group (Fig. 2D).

There was no statistically significant difference in the number of circulating tumor cells between the CEACAM1 kd group and the Luc control group (p = 0.756; Fig. 2E).

### IGFBP7 and Latexin are the most up- and down-regulated genes in the CEACAM1 kd tumors

**Figure 3:**
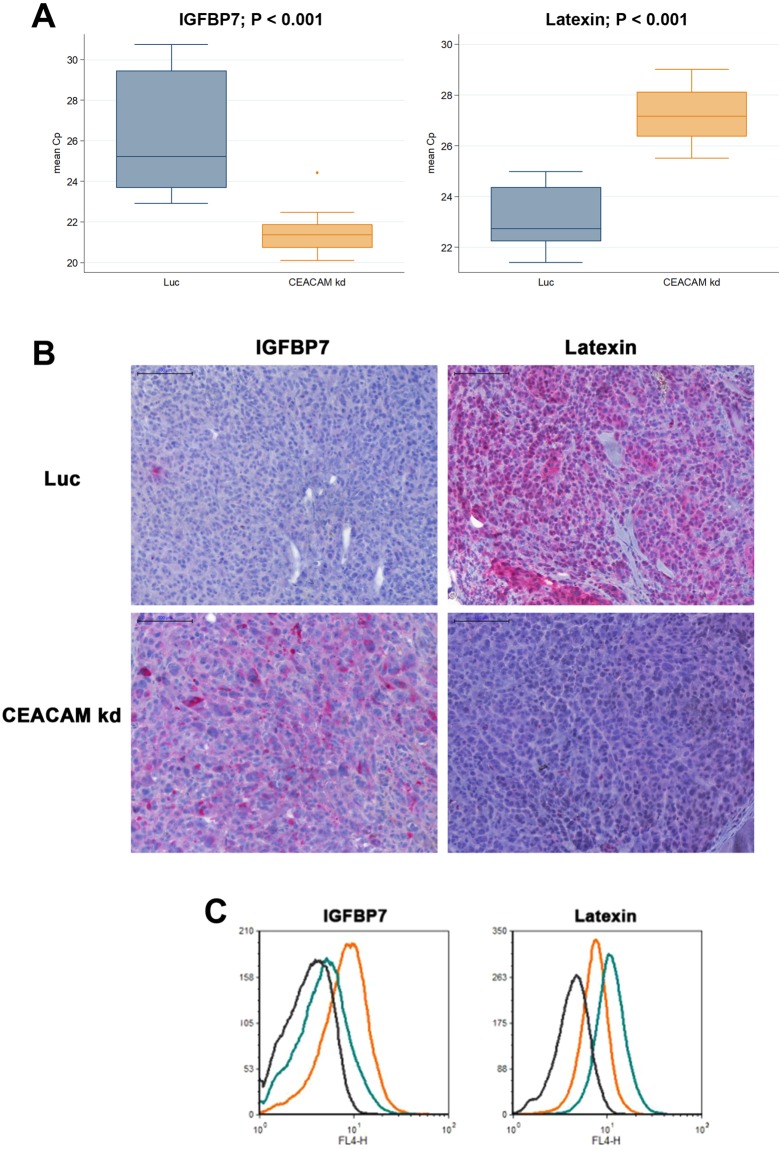
IGFBP7 expression is up- and Latexin expression is downregulated in the xenograft melanoma induced by CEACAM1 kd cells. (**A**) IGFBP7 and Latexin expression are significantly regulated on the transcriptional level as determined by qRT-PCR with template cDNA derived from RNA from MeWo tumors (human melanoma cell line) subcutaneously grown in scid mice. Please note that lower Cp values mean higher levels of expression: IGFBP7 expression is significantly higher in MeWo CEACAM1 kd than in MeWo Luc control tumors whereas latexin expression is significantly lower in the MeWo CEACAM1 kd tumors. Each experiment was performed twice with 5 replicates each time. (**B**) Immunohistochemical staining of paraffin embedded tumor tissue (xenograft MeWo tumors in scid mice) for IGFBP7 and latexin demonstrated respective up- and downregulation of these two proteins. Little to no IGFBP7 (in red) could be shown in the MeWo Luc tumors (upper left panel) whereas high IGFBP7 was detected in the MeWo CEACAM1 kd tumors (lower left panel). Vice versa, high latexin expression could be detected in the MeWo Luc tumors (upper right panel) and little to no latexin expression in the CEACAM1 kd tumors (lower right panel). (**C**) IGFBP7 and latexin expression are up- and downregulated, respectively, on the cell culture level in MeWo CEACAM1 kd cells compared with MeWo Luc cells: Flow cytometric determination of intracellular IGFBP7 and latexin in MeWo Luc (teal curves) and MeWo CEACAM1 kd cells (orange curves) with corresponding isotype controls (black curves).

In Affymetrix® whole genome microarrays with RNA from the xenograft MeWo tumors (Luc and CEACAM1 kd) the expression total of 703 genes was found to be significantly altered with a fold change of at least 1.5 (SLR of 0.6/−0.6) between the groups. Of these, 257 were up- and 446 down-regulated (Supplementary Table 1). One of the latter genes (and only CEA family member that was found to be definitely expressed at all) was CEACAM1 (fold change of −2.0 or SLR = −1.0). Other CEACAM family members potentially affected by the shRNA were not significantly regulated (Supplementary Table 2). As both the most up-regulated (Insulin-like growth factor-binding protein 7, IGFBP7, fold change 10.6 or SLR = 3.4) and the most down-regulated gene (Latexin, LXN, fold change 73.5 or SLR = −6.2) have been implied to have important roles in malignant progression (see Discussion below), these two candidates were chosen to exemplarily verify the array results. The respective up- and down-regulation of IGFBP7 and LXN in the MeWo CEACEM1 knockdown tumors compared with the Luc control tumors could be verified by quantitative real-time PCR where IGFBP7 and LXN were found to be up and down regulated, respectively (−4.8, 95%-CI [−7.0;−2.5], p < 0.001 and +4.2 [3.0;5.3], p < 0.001, respectively; GAPDH as control; Fig. 3A). Additionally, the results from the array could be verified at the protein level by immunohistochemistry for the xenograft tumors: Strong staining for LXN was observed in the Luc control tumors only, whereas strong IGFBP7 staining was exclusively observed in the CEACAM1 kd tumors (Fig. 3B).

The regulation of IGFBP7 and LXN upon CEACAM1 kd could also be demonstrated in cultured MeWo cells by flow cytometry (intracellular staining): The fluorescence signal for LXN was only 31% in the CEACAM1 kd cells (signal above isotype background MeWo CEACAM1 kd: 2.2; MeWo Luc: 7.02) whereas the signal for IGFBP7 was 296% in the CEACAM1 kd cells (signal above isotype background MeWo CEACAM1 kd: 5.48; MeWo Luc: 1.85; Fig. 3C). IGFBP7 and LXN were also up- and downregulated, respectively, in the corresponding lung metastases of the xenograft model (Supplementary Fig. 2).

### CEACAM1 knockdown interferes with the melanoma cells’ network of EMT genes

In the CEACAM knockdown tumors, genes reported to be involved in the epithelial to mesenchymal transition (EMT) network of melanoma cells (see Discussion below) were significantly down- or upregulated: BRAF (−1.5 fold), CDH2 (N-cadherin, −1.6 fold), FOSL1 (or Fra-1, −1.7 fold), NRAS (−1.6 fold), SNAI2 (or SLUG, +2.0 fold) and TWIST1 (−1.6 fold).

### High CEACAM1 expression indicates poor survival of melanoma patients

**Figure 4:**
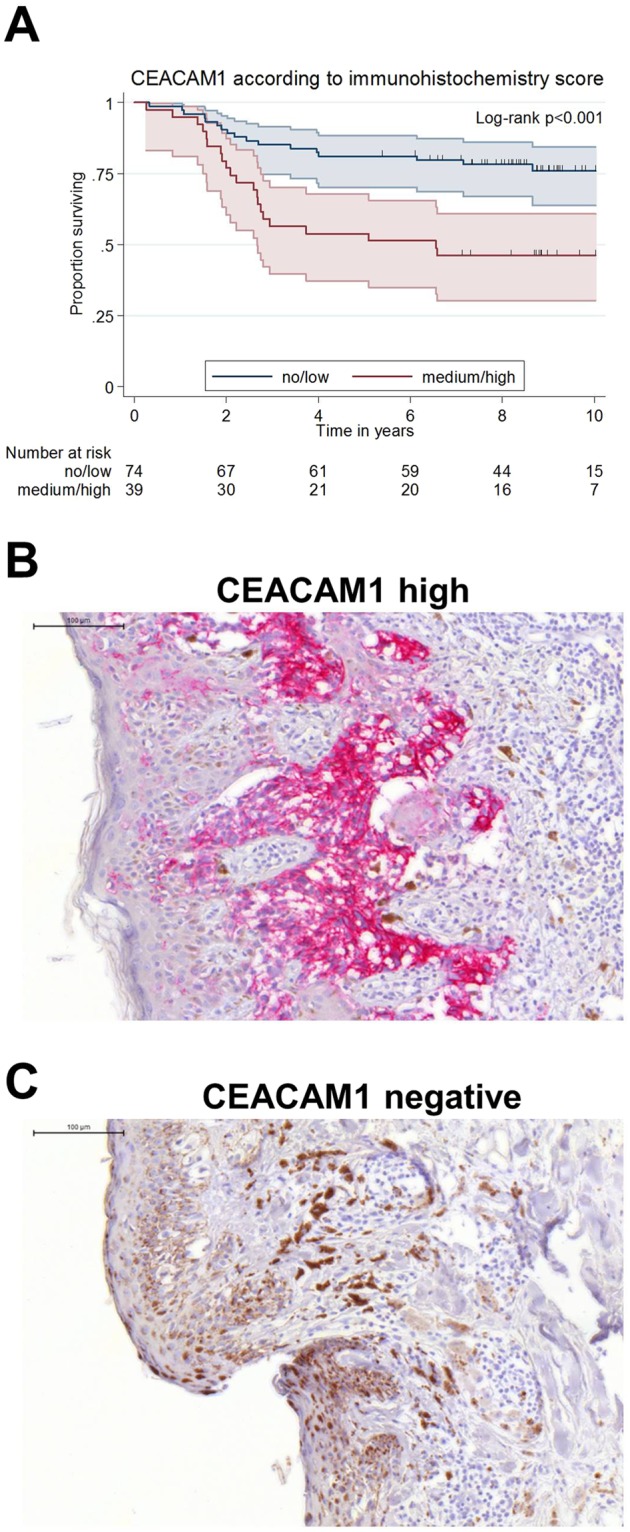
CEACAM1 expression in melanoma is inversely correlated with patient survival. (**A**) Kaplan-Meier overall-survival analysis (OS): CEACAM1 expression in melanoma samples was visualized by CEACAM1 specific immunohistochemistry in melanoma samples from 113 patients. Patients were grouped as CEACM1 “no/low” staining or CEACAM1 “medium/high” staining. CEACAM “no/low” patients show a significantly longer overall survival than CEACAM “medium/high” patients (p < 0.001). (**B**) A melanoma with high CEACAM1 expression: Immunohistochemical staining of paraffin embedded patient tissue for CEACAM1. (**C**) A CEACAM1 negative melanoma: Immunohistochemical staining of paraffin embedded patient tissue for CEACAM1.

CEACAM1 expression in melanoma of 113 patients could be investigated by staining for CEACAM1 using immunohistochemistry. When the patients are grouped according to their CEACAM1 immunohistochemistry score (high or medium (n = 39) against low or negative (n = 74)), prognosis for CEACAM1 low patients is significantly better (p < 0.001, Fig. 4A). The 5-year survival is 81.1% (95%-CI [70.2;88.3]) for the CEACAM1 low patients against 53.9% (95%-CI [37.2;67.9]) for the CEACAM1 high patients. For an isotype control for CEACAM1 staining please see Supplementary Fig. 3.

### Latexin correlates with CEACAM1 expression in melanoma and indicates poor survival

**Figure 5:**
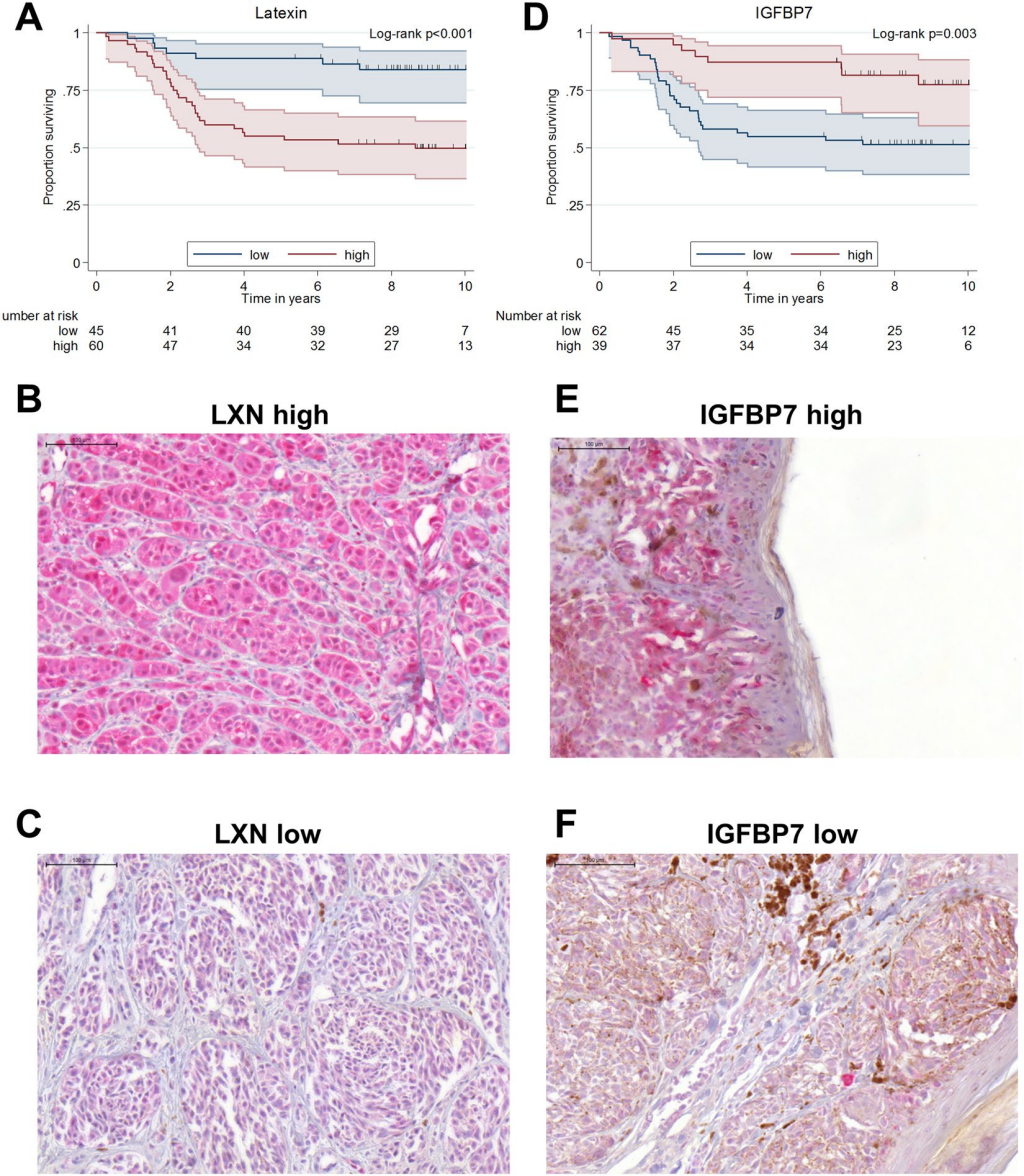
Latexin expression in melanoma is inversely correlated with patient survival whereas IGFBP7 expression in melanoma is positively correlated with patient survival. (**A**) Kaplan-Meier overall-survival analysis (OS): Latexin (LXN) expression in melanoma samples was visualized by LXN specific immunohistochemistry in melanoma samples from 105 patients. Patients were grouped as LXN 0–1 (no or low LXN staining) or LXN 2–3 (medium or high LXN staining). LXN low patients show a significantly longer overall survival than LXN high patients (p < 0.001). (**B**) A melanoma with high LXN expression: Immunohistochemical staining of paraffin embedded patient tissue for LXN. (**C**) A melanoma with low LXN expression: Immunohistochemical staining of paraffin embedded patient tissue for LXN. (**D**) Kaplan-Meier overall-survival analysis (OS): IGFBP7 expression in melanoma samples was visualized by IGFBP7 specific immunohistochemistry in melanoma samples from 101 patients. Patients were grouped as IGFBP7 low (no or low IGFBP7 staining) or IGFBP7 high (high or very high IGFBP7 staining). IGFBP7 high patients show a significantly higher overall survival than IGFBP7 low patients (p = 0.003). (**E**) A melanoma with high IGFBP7 expression: Immunohistochemical staining of paraffin embedded patient tissue for IGFBP7. (**F**) A melanoma with low IGFBP7 expression: Immunohistochemical staining of paraffin embedded patient tissue for IGFBP7.

LXN expression could be investigated by immunohistochemistry in melanoma from 105 patients of the same cohort from which the results for CEACAM1 expression were obtained. According to the results from the xenograft experiment, we hypothesized that melanoma cells with high LXN expression also have a higher metastatic potential than LXN low cells. Of these samples, 45 displayed no or low LXN expression (score of 0 or 1; group LXN low) and 60 displayed medium or high LXN expression (score 2 or 3; group LXN high). High LXN expression in the melanoma specimens correlated with shortened patient survival (age-adjusted HR 4.0, 95%-CI [1.8;9.1]; p = 0.001), with a 5-year survival of 88.9% (95%-CI [75.3;95.2]) in the LXN low patients in comparison with 55.0% (95%-CI [41.6;66.5]) for the LXN high patients (Fig. 5A).

High expression of CEACAM1 in the melanoma samples correlated with high LXN expression (ϕ = 0.37, p < 0.001) and also CEACAM1 staining in a certain area of an individual melanoma often correlated with LXN staining intensity in the same area (serial sections, e.g. Supplementary Fig. 5). For an isotype control for LXN staining please see Supplementary Fig. 3.

### High IGFBP7 expression indicates prolonged survival of melanoma patients

IGFBP7 expression could be investigated by immunohistochemistry in melanoma from 101 patients of the same cohort as above. Following the results from the xenograft experiment, we hypothesized that melanoma cells with no or weak IGFBP7 expression have a higher metastatic potential than IGFBP7 high cells. Therefore, all melanoma samples were classified into two groups: IGFBP7 high, meaning that nearly all tumor tissue displayed moderate or high IGFBP7 staining or IGFBP7 low, with at least 20% tumor tissue negative or only weakly positive for IGFBP7. 62 samples were classified into the IGFBP7 low group, 39 samples were regarded as IGFBP7 high. Patients whose melanoma displayed the IGFBP7 high staining pattern had a better prognosis (longer survival) in comparison to the IGFBP7 low patients (age-adjusted HR 0.32, 95%-CI [0.15;0.71]; p = 0.005). 5-year survival of IGFBP7 high patients was 87.2% (95%-CI [71.9;94.5]) in comparison with 54.8% (95%-CI [41.7;66.2]) for the IGFBP7 low patients (Fig. 5D).

In the melanoma samples, high expression of CEACAM1 did not correlate with low IGFBP7 expression and low IGFBP7 expression did not correlate with high LXN expression (ϕ = −0.01, p = 0.911 and ϕ = 0.03, p = 0.781, respectively). CEACAM1 staining in a certain area of an individual melanoma sometimes correlated with IGFBP7 staining intensity in the same area, but more often did not (serial sections, e.g. Supplementary Fig. 6). For an isotype control for IGFBP7 staining please see Supplementary Fig. 3.

### Expression of TWIST in the melanoma samples

**Figure 6:**
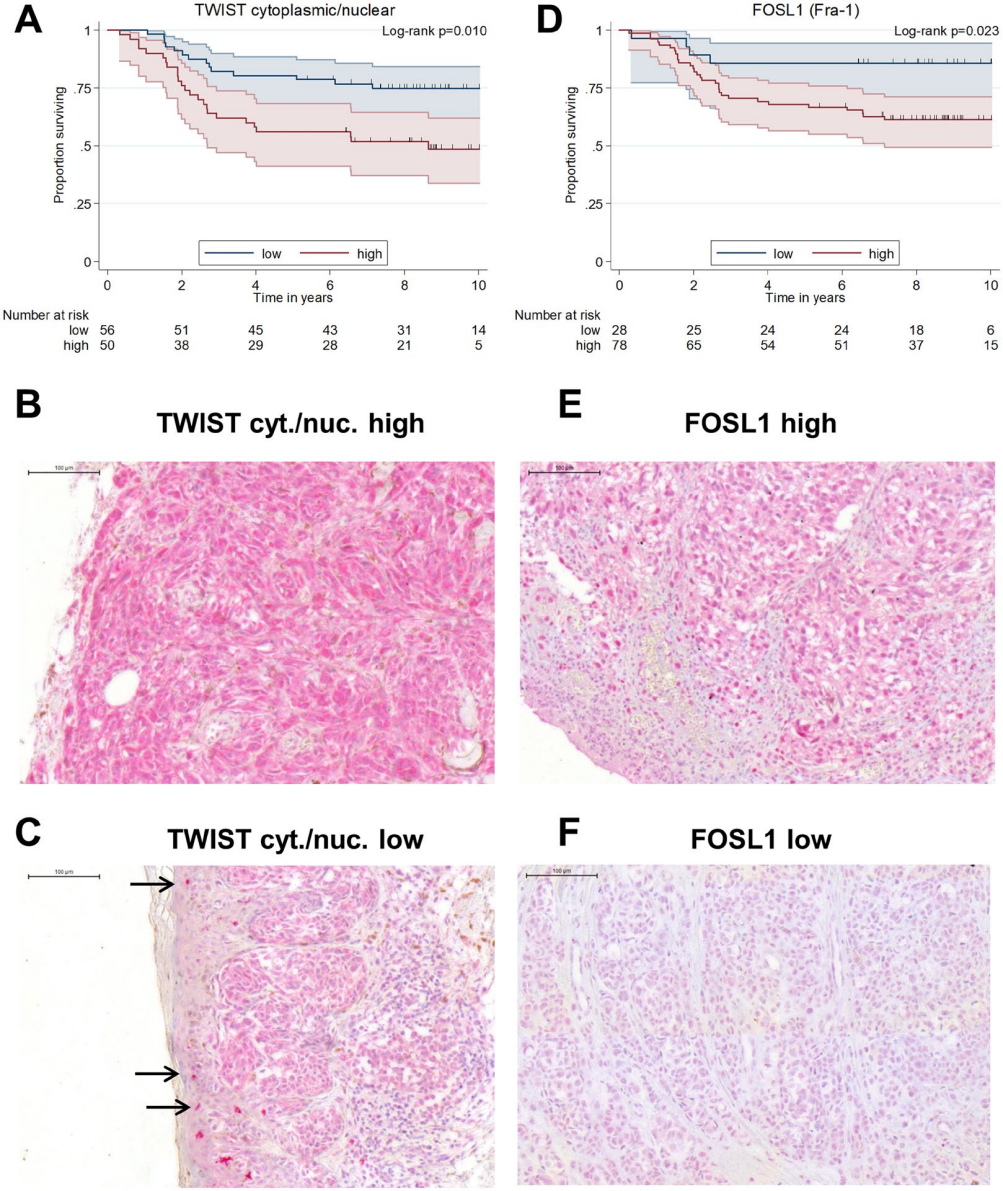
TWIST cytoplasmic/nuclear (cyt./nuc.) expression/location and FOSL1 (Fra-1) expression in human melanoma are correlated with patient survival. (**A**) Kaplan-Meier overall-survival analysis (OS): TWIST expression in melanoma samples was visualized by TWIST specific immunohistochemistry in melanoma samples from 106 patients. Patients were grouped as TWIST cyt./nuc. low (cyt./nuc. TWIST ≤ 2) or TWIST high (cyt./nuc. TWIST > 2). TWIST cyt./nuc. low (≤2) show a significantly longer overall survival than TWIST cyt./nuc. high (>2) patients (p = 0.010). (**B**) A melanoma with high TWIST cyt./nuc. expression: Immunohistochemical staining of paraffin embedded patient tissue for TWIST. (**C**) A melanoma with low TWIST cyt./nuc. expression: Immunohistochemical staining of paraffin embedded patient tissue for TWIST. Arrows indicate highly TWIST positive nuclei in normal skin epidermis. (**D**) Kaplan-Meier overall-survival analysis (OS): FOSL1 expression in melanoma samples was visualized by FOSL1 specific immunohistochemistry in melanoma samples from 106 patients. Patients were grouped as FOSL1 0–1 (no or low FOSL staining) or FOSL1 2–3 (medium or high FOSL1 staining). FOSL1 low patients show a significantly longer overall survival than FOSL high patients (p = 0.023). (**E**) A melanoma with high FOSL1 expression: Immunohistochemical staining of paraffin embedded patient tissue for FOSL1. (**F**) A melanoma with low FOSL1 expression: Immunohistochemical staining of paraffin embedded patient tissue for FOSL1.

In the patients’ melanoma specimens, single cells in the stratum basale of the epidermis of the normal skin within the samples showed nuclear staining for TWIST (presumably epidermal stem cells). Notably, the staining intensity of these normal epidermal stem cells markedly differed between patients. The melanoma cells in the samples also showed nuclear TWIST with staining intensities that could be lower, as high as or even higher (the latter being seldom, 9 cases) than the staining intensities of the epidermal stem cells of the normal skin tissue of the same sample. Nuclear TWIST staining was classified accordingly as 1 (lower than the stem cells), 2 (as high as the stem cells) or 3 (higher than the stem cells). Additionally, the melanoma cells always showed low to very high cytoplasmic staining for TWIST. Accordingly, cytoplasmic TWIST in the melanoma cells was classified as 1 (low), 2 (medium), 3 (high) and 4 (very high). Nuclear and cytoplasmic TWIST staining could be obtained from the melanoma samples of 106 patients. Regarding the samples’ melanoma cells, a high quotient of cytoplasmic/nuclear Twist (groups were: low [cytoplasmic-to-nuclear Twist ratio ≤ 2] and high [cytoplasmic-to-nuclear Twist ratio > 2]) was associated with a higher risk for death in comparison with low proportion of cytoplasmic Twist (age-adjusted HR 2.3, 95%-CI [1.2;4.3]; p = 0.013). Better prognosis for TWIST cytoplasmic/nuclear low patients comes with a 5-year survival of 80.4% (95%-CI [67.3;88.6]) compared with 56.0% (95%-CI [41.2;68.4]) 5–year survival for the TWIST cytoplasmic/nuclear high patients (Fig. 6A). High expression of CEACAM1 in the melanoma samples correlated with high cytoplasmic/nuclear Twist (ϕ = 0.20, p = 0.049) and also CEACAM1 staining in a certain area of an individual melanoma often correlated with cytoplasmic/nuclear TWIST staining intensity in the same area (serial sections, e.g. Supplementary Fig. 7). For an isotype control for TWIST staining please see Supplementary Fig. 4. Additionally, cytoplasmic/nuclear Twist was low in the CEACAM1 kd lung metastases of the xenograft model (Supplementary Fig. 2).

### Expression of FOSL1 (Fra-1) in the melanoma samples

As FOSL1 (Fra-1) links the BRAF/NRAS pathway to TWIST (see Discussion below), we chose to verify the microarray results from the xenograft experiment in the patients’ melanoma samples by staining for FOSL1.

FOSL1 expression could be investigated by immunohistochemistry in melanoma from 106 patients. Of these samples, 28 displayed no or low FOSL1 expression (score of 0 or 1; group FOSL1 low) and 78 displayed medium or high FOSL expression (score 2 or 3; group FOSL1 high). High FOSL expression in the melanoma was correlated with shortened patient survival (age-adjusted HR 3.2, 95%-CI [1.1;9.1]; p = 0.029) with a 5-year survival of 68.0% (95%-CI [56.4;77.1]) in comparison to 85.7% (95%-CI [66.3;94.4]) for the FOSL1 low patients (Fig. 6D). High expression of CEACAM1 in the melanoma samples correlated with high FOSL1 (ϕ = 0.36, p < 0.001) and also CEACAM1 staining in a certain area of an individual melanoma often correlated with FOSL1 staining intensity in the same area (serial sections, e.g. Supplementary Fig. 8). For an isotype control for FOSL1 staining please see Supplementary Fig. 4. FOSL1 expression was also low in the CEACAM1 knockdown lung metastases of the xenograft model (Supplementary Fig. 2).

### Combining above markers in a multivariate model for prognosis of survival

The markers CEACAM1, FOSL1, IGFBP7, LXN and cytoplasmic/nuclear TWIST were used as predictors in a multivariate Cox model (adjusted for age) for the prognosis of the melanoma patients’ survival. After stepwise selection procedure IGFBP7, LXN and cyt./nuc. TWIST remained in the model. High IGFBP7 (HR 0.31, 95%-CI [0.13;0.72]; p = 0.006), low cyt./nuc. Twist (HR 0.43, 95%-CI [0.21;0.86]; p = 0.018) and low LXN (HR 0.32, 95%-CI [0.14;0.75]; p = 0.008) were associated with a lower risk of dying.

## Discussion

In the cohort examined in our study a high CEACAM1 expression in melanoma was negatively correlated with survival which corresponds well with the literature^12–14^, verifying this correlation in an additional cohort of patients. Multivariate analysis revealed other more suitable prognostic markers for death (IGFBP7, LXN and cyt./nuc.TWIST), of which LXN and TWIST were, however, correlated with the regulation of CEACAM1. Elimination of CEACAM1 as an additional prognostic marker in the multivariate analysis is probably due to the strong regulatory interplay of CEACAM1 which was also indicated by the whole genome expression array analysis (703 significantly regulated genes including IGFBP7, LXN and TWIST). CEACAM1 association with short survival was reflected in our xenograft model of primary tumor growth and spontaneous metastasis in which both were significantly reduced by CEACAM1 knockdown increasing the animals’ survival. To our knowledge this is the first direct *in vivo* demonstration in a spontaneous metastasis model that CEACAM1 is functionally involved in melanoma metastasis formation. As no functional lymphocytes are present in scid mice, the observed effect on metastasis (and tumor growth) in this study cannot be T cell-dependent as could be assumed based on a previous study suggesting that CEACAM-1 depletion results in T cell activation^14^. This makes CEACAM1 inhibition an interesting option for combination therapy with immune checkpoint inhibitors (e.g. PD-1/PD-1L) as it would target additional factors/pathways that enable the melanoma cells to metastasize. It could also be an alternative for patients with melanoma resistant to immune checkpoint inhibition. Additionally, the results from our xenograft model with a BRAF wildtype human melanoma cell line show that the effect is obviously independent of mutated BRAF. CEACAM inhibition could thus also be an alternative to BRAF-inhibition or be combined with BRAF inhibitors, especially as CEACAM1 knockdown additionally decreased BRAF (and NRAS) expression in the xenograft tumors.

Remarkably, the *in vitro* results from cell culture (increased *in vitro* proliferation and migration in the CEACAM1 kd) could not predict the melanoma cells’ *in vivo* behavior (decreased tumor growth and metastasis in the CEACAM1 kd) whereas the xenograft model reflected the clinical situation quite well (high CEACAM1 expression is associated with high metastatic potential in xenograft models and with poor survival of patients, see also analyses discussed below). This strongly argues for *in vivo* models as experimental ultima ratio as only these can model the entire metastatic cascade.

Microarray analysis revealed that CEACAM1 is strongly involved in the melanoma cells’ network of EMT associated genes. Remarkably, in the CEACAM1 kd tumors BRAF and NRAS expressions are downregulated, accompanied by upregulation of SNAI2 (SLUG) and downregulation of TWIST, N-cadherin and FOSL1 (Fra-1) of which the latter links TWIST to the BRAF/NRAS pathway^25^. To exemplarily verify the results from the xenograft tumors, patient melanoma samples were stained for TWIST and FOSL1 revealing a correlation between cytoplasmic to nuclear TWIST and CEACAM1 expression and also between FOSL1 and CEACAM1 expression.

In the gene expression microarrays, IGFBP7 – a potent tumor (and probably metastasis) suppressor in melanoma^26,27^ – was the gene with the highest upregulation in expression in the CEACAM1 knockdown tumors. In the melanoma patient samples, high CEACAM1 expression did not correlate with low IGFBP7 expression which might be due to the fact that the IGFBP7 promotor is silenced (methylated) in most melanoma with BRAF mutation which comprise roughly half of all cases of melanoma^27^ in which low CEACAM1 expression would still not lead to an increase in IGFBP7. However, as the cell line used in this study is BRAF wildtype it is still possible that CEACAM1 signaling affects IGFBP7 expression in certain subsets of patients (e.g. patients with BRAF wildtype melanoma).

LXN (Latexin) has been described as a putative tumor suppressor in melanoma^28^ as its expression was downregulated in most melanoma cells compared with melanocytes and normal skin in 50% of melanoma samples and its promotor was found to be methylated in most melanomas and melanoma cell lines. In the study cited above, most melanoma samples showed only low immunohistochemical LXN staining. However, LXN staining was done for a total of 11 patient samples only. Additionally, melanoma cells with forced LXN expression showed reduced proliferation. Apart from the latter result (as increased *in vitro* proliferation was indeed found for the LXN low CEACAM1 kd MeWo cells), the data from this study do not support the classification of LXN as a tumor or even a metastasis suppressor. The LXN low CEACAM1 kd MeWo tumors displayed reduced *in vivo* growth and metastasis in the spontaneous metastasis xenograft model and survival for patients with medium or high LXN melanoma on protein level (total of 105 samples) was significantly shortened. The most important question concerning the expression of a particular gene in melanoma is its role in metastasis formation. The data presented here together with the previous study^28^ certainly argue for further studies to carefully evaluate the role of LXN in melanoma in more detail, especially as of the three markers (IGFBP7, LXN and cyt./nuc.TWIST) which significantly contributed to prognosis in the selected model LXN would be the easiest to interfere with e.g. by pharmacological inhibition. As high expression of IGFBP7 is positively correlated with survival one would have to find a way to upregulate this tumor suppressor. According to the data presented here this could probably be achieved by blocking CEACAM1 in some melanoma subsets (e.g. BRAF wildtype with high CEACAM1 expression). Concerning TWIST, an interesting question is certainly if simply blocking this transcription factor would be beneficial or if its expression has to be to “normalized” instead by shifting the balance of its location from cytoplasmic back to nuclear.

Taken together, the data presented here argue for CEACAM1 as a potential therapeutic target especially for combination therapies as the effects of reducing CEACAM1 signaling are apparently independent of the BRAF mutational status and (at least in part) of T cell responses. Thus inhibition of CEACAM1 signaling might prove highly effective combined with BRAF and immune checkpoint inhibition.

### Ethical Approval and Informed Consent

The xenograft experiment was recommended and supervised by the institutional animal welfare officer and approved by the local licensing authority (Behörde für Soziales, Familie, Gesundheit, Verbraucherschutz; Amt für Gesundheit und Verbraucherschutz; Billstr. 80, D-20539 Hamburg, Germany). All methods were performed in accordance with the relevant guidelines and regulations by the local authorities. For this study, tissue samples were recruited following written informed consent of the patients. Tissue collection was approved by the local institutional review board of the Westphalian Wilhelms University in Münster, Germany.

## Electronic supplementary material

Supplementary Information
