# Supplementary material for: CEACAM1 promotes melanoma metastasis and is involved in the regulation of the EMT associated gene network in melanoma cells
Source: Sci Rep. 2018 Aug 8;8:11893. doi: 10.1038/s41598-018-30338-4 (PMC6082866; doi:10.1038/s41598-018-30338-4)
Supplement: Supplementary file 1 — Supplementary Information [file 41598_2018_30338_MOESM1_ESM.pdf]

Supplementary Information

CEACAM1 promotes melanoma metastasis and is involved in the regulation of the EMT associated gene network in melanoma cells

Daniel Wicklein[1], Benjamin Otto[2,3], Anna Suling[4], Eva Elies[1], Georg Lüers[1], Tobias Lange[1], Susanne Feldhaus[1], Hanna Maar[1], Jennifer Schröder-Schwarz[1], Georg Brunner[5], Christoph Wagener[6*], Udo Schumacher[1*]

* These authors equally contributed to this work.

1 Institute of Anatomy and Experimental Morphology, University Cancer Center, University Medical-Center Hamburg-Eppendorf, Hamburg, Germany

2 Eppendorf AG, Hamburg, Germany

3 Department of Medicine I, University Medical Center Hamburg-Eppendorf, Hamburg, Germany

4 Department of Medical Biometry and Epidemiology, University Medical Center Hamburg-Eppendorf, Hamburg, Germany

5 Department of Cancer Research, Fachklinik Hornheide, Münster, Germany. Present address: NeraCare GmbH, Bönen, Germany

6 Center for Diagnostics, University Medical Center Hamburg-Eppendorf, Hamburg, Germany

Corresponding author:

Daniel Wicklein, PhD,
Institute of Anatomy and Experimental Morphology, University Cancer Center,
University Medical Center Hamburg-Eppendorf

Martinistrasse 52
20246 Hamburg

Phone: (+)49-40-7410-52591
FAX: (+)49-40-7410-55427

Email: d.wicklein@uke.de

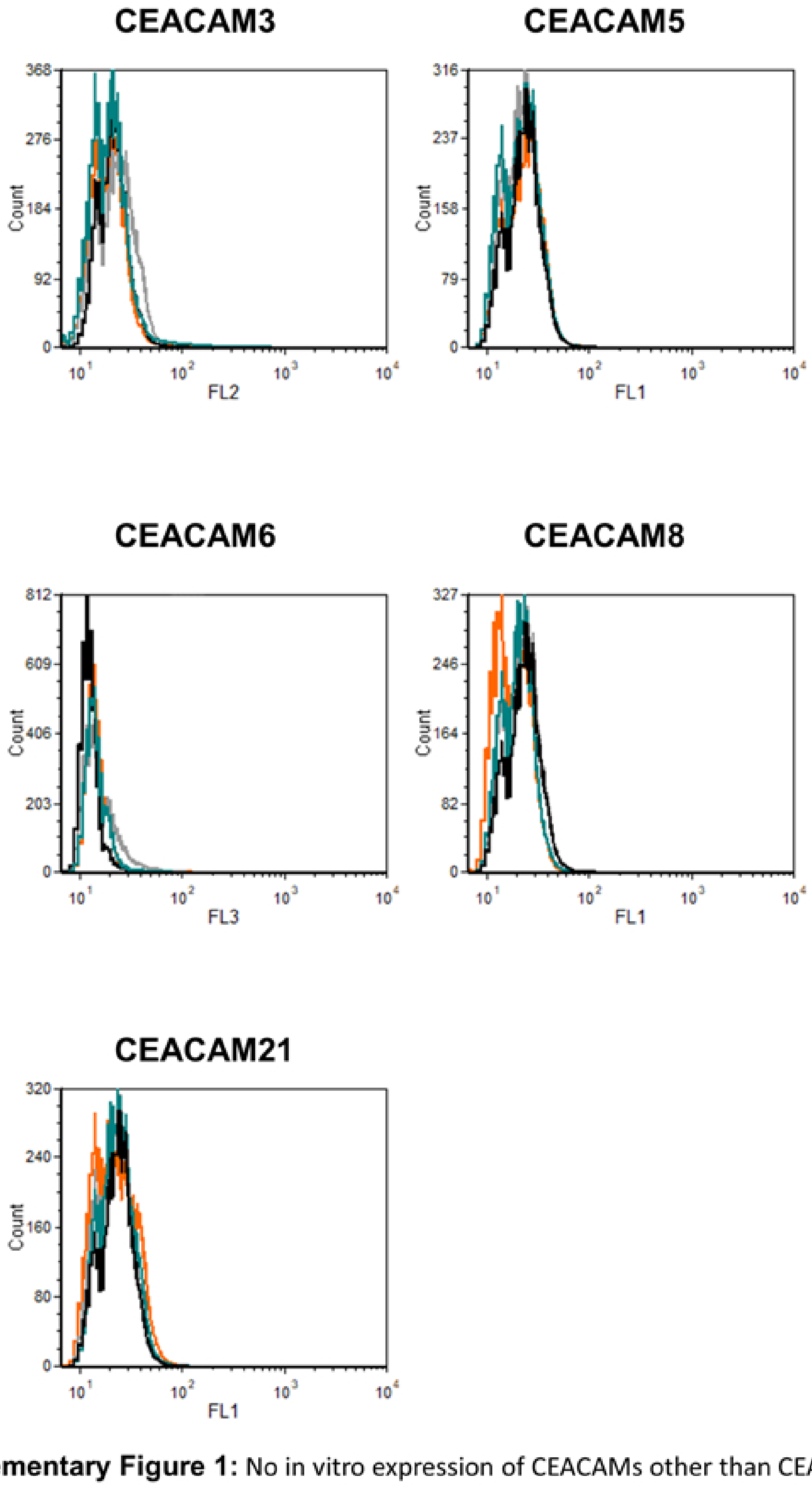

Supplementary Figure 1: No in vitro expression of CEACAMs other than CEACAM1. Flow Cytometry for fixed and permeabilized cells. Staining of MeWo CEACAM1 kd (orange curve, respective isotype control in grey) and MeWo Luc (teal, respective isotype control in black) for CEACAM3, 5, 6, 8 and 21, respectively.

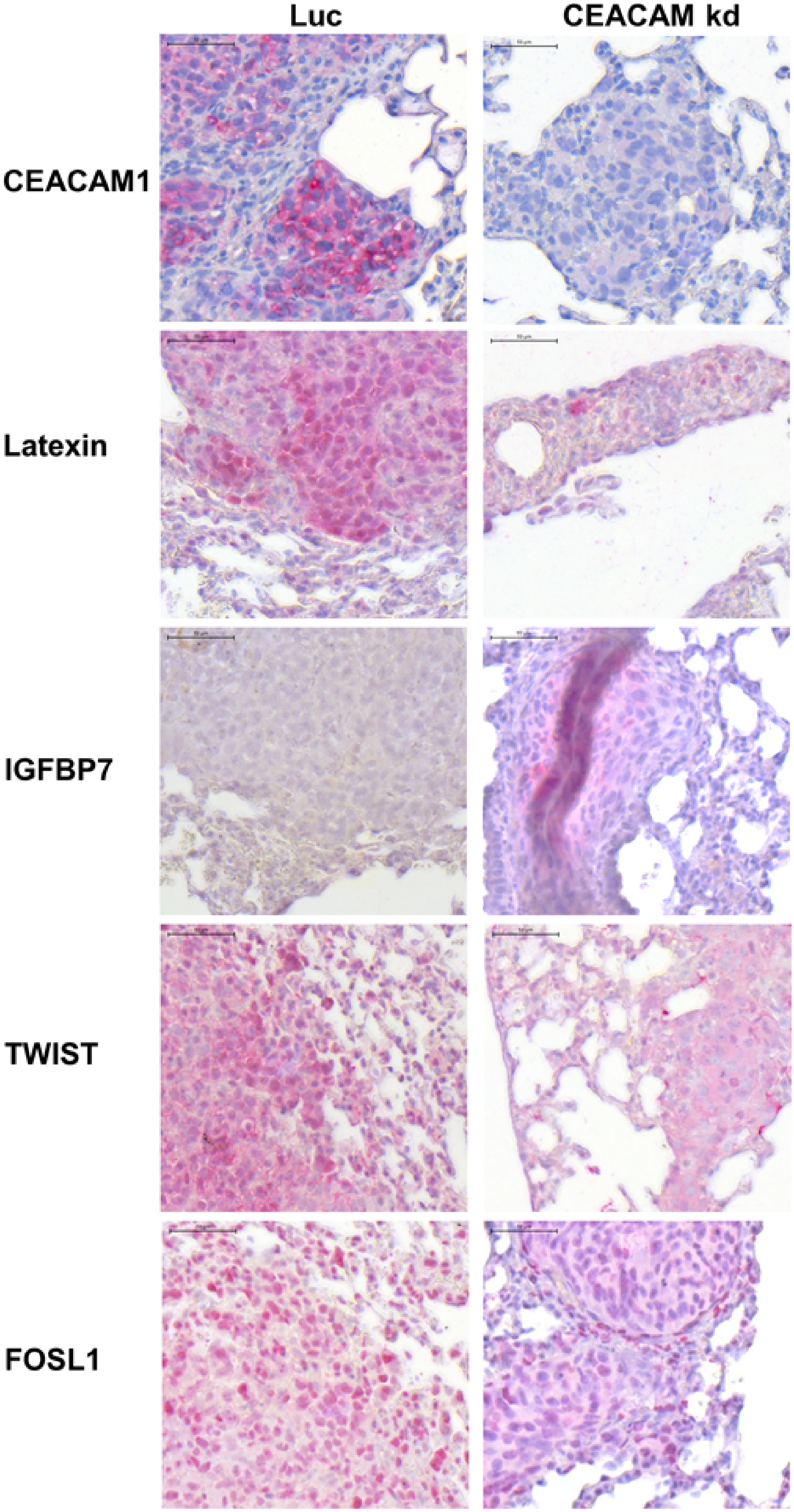

Supplementary Figure 2 Comparison of CEACAM1, Latexin, IGFBP7, TWSIT1 and FOSL1 expression in the xenograft lung metastases. Immunohistochemical staining of paraffin embedded mouse lung tissue (xenograft subutaenous primary MeWo tumors in scid mice) for CEACAM1, Latexin, IGFBP7, TWIST and FOSL1 demonstrated respective up- or downregulation of these proteins in the lung metastases induced by CEACAM kd or Luc control tumors.

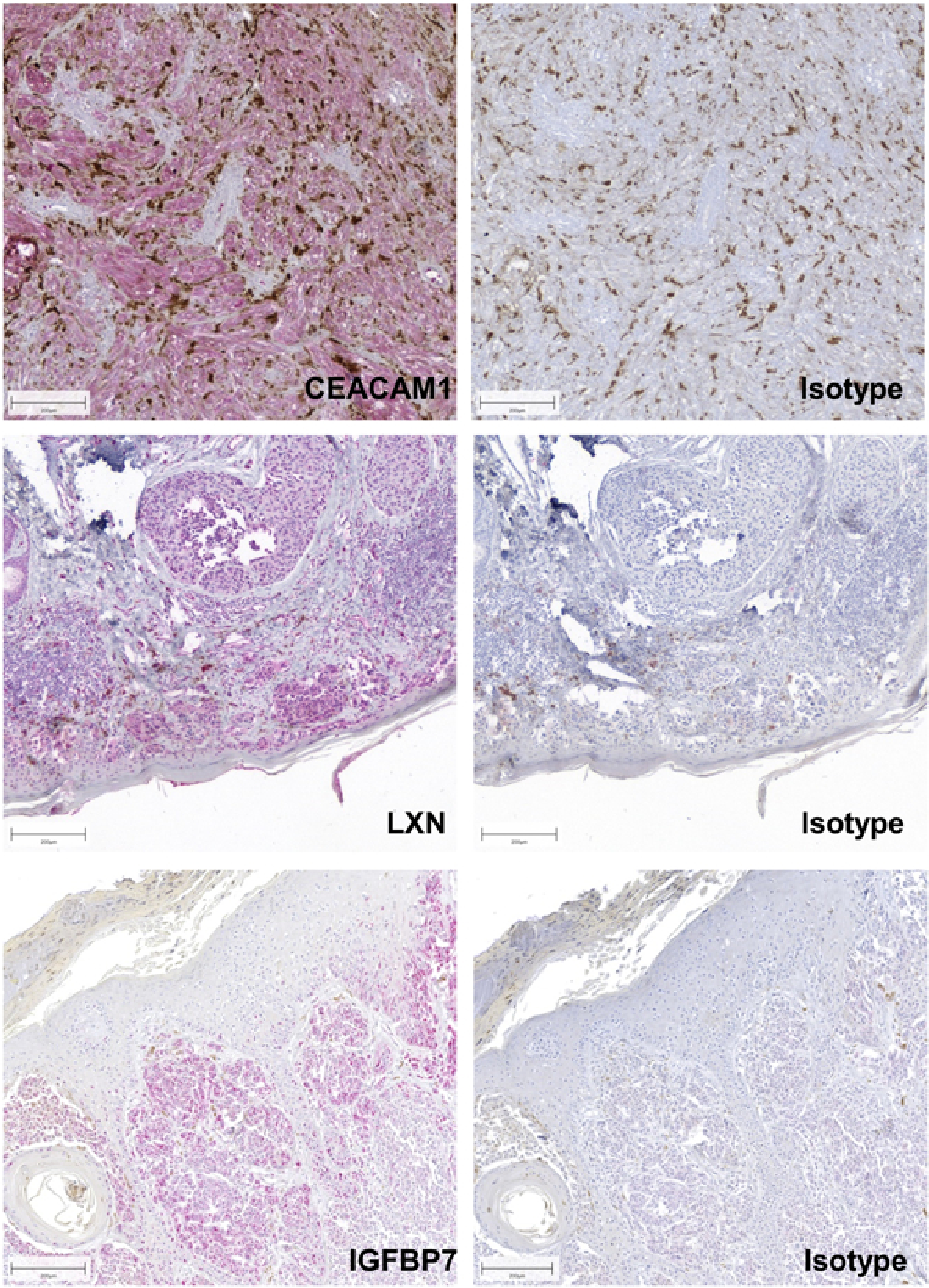

Supplementary Figure 3: Immunohistochemical staining of paraffin embedded patient melanoma tissue for CEACAM1 (upper panel), Latexin (LXN, middle panel) and IGFBP7 (lower panel) and the respective Isotype Controls.
scale bar: 200µm

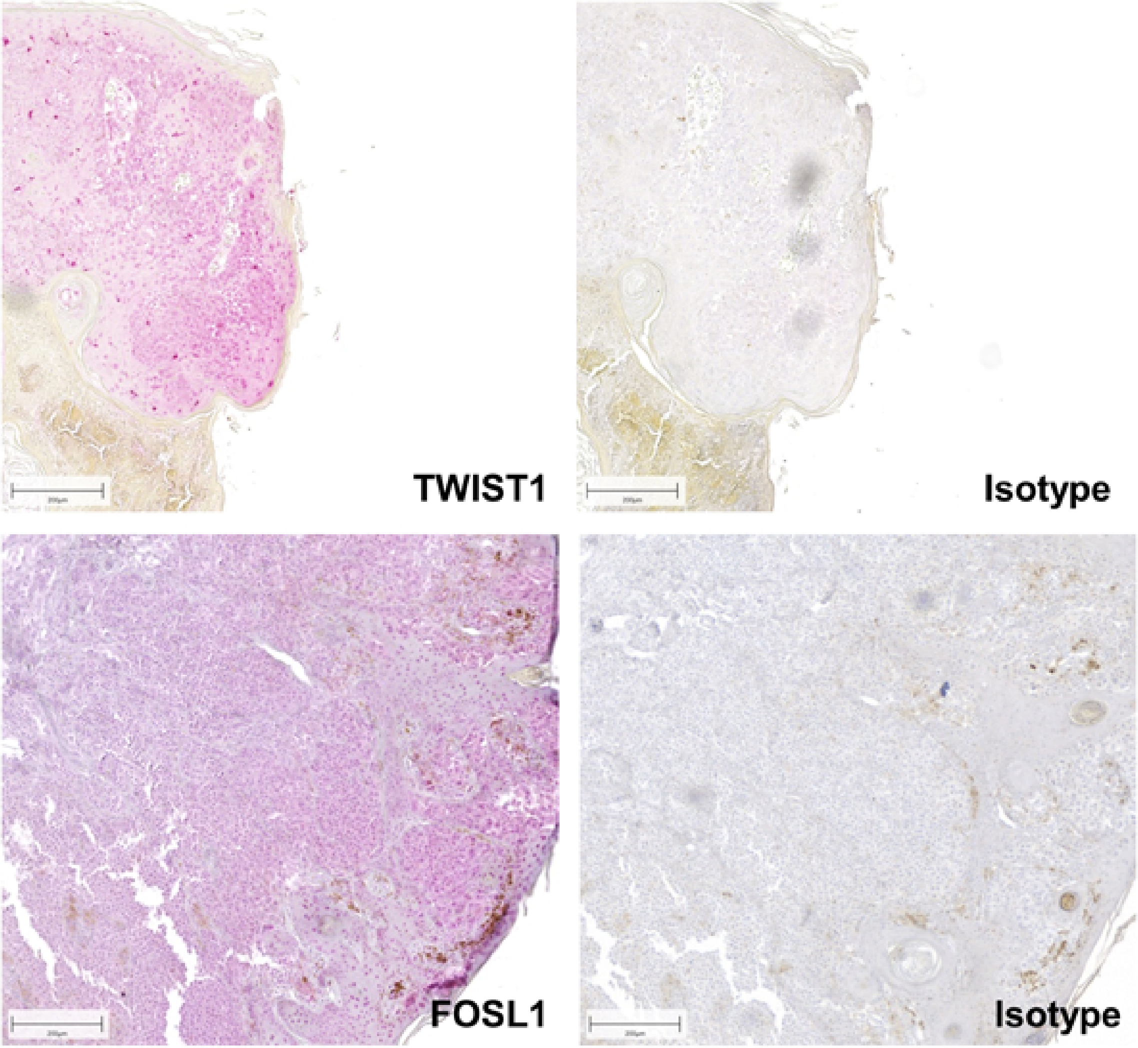

Supplementary Figure 4: Immunohistochemical staining of paraffin embedded patient melanoma tissue for TWIST1 (upper panel) and Fra-1(lower panel) and the respective Isotype Controls. scale bar: 200µm

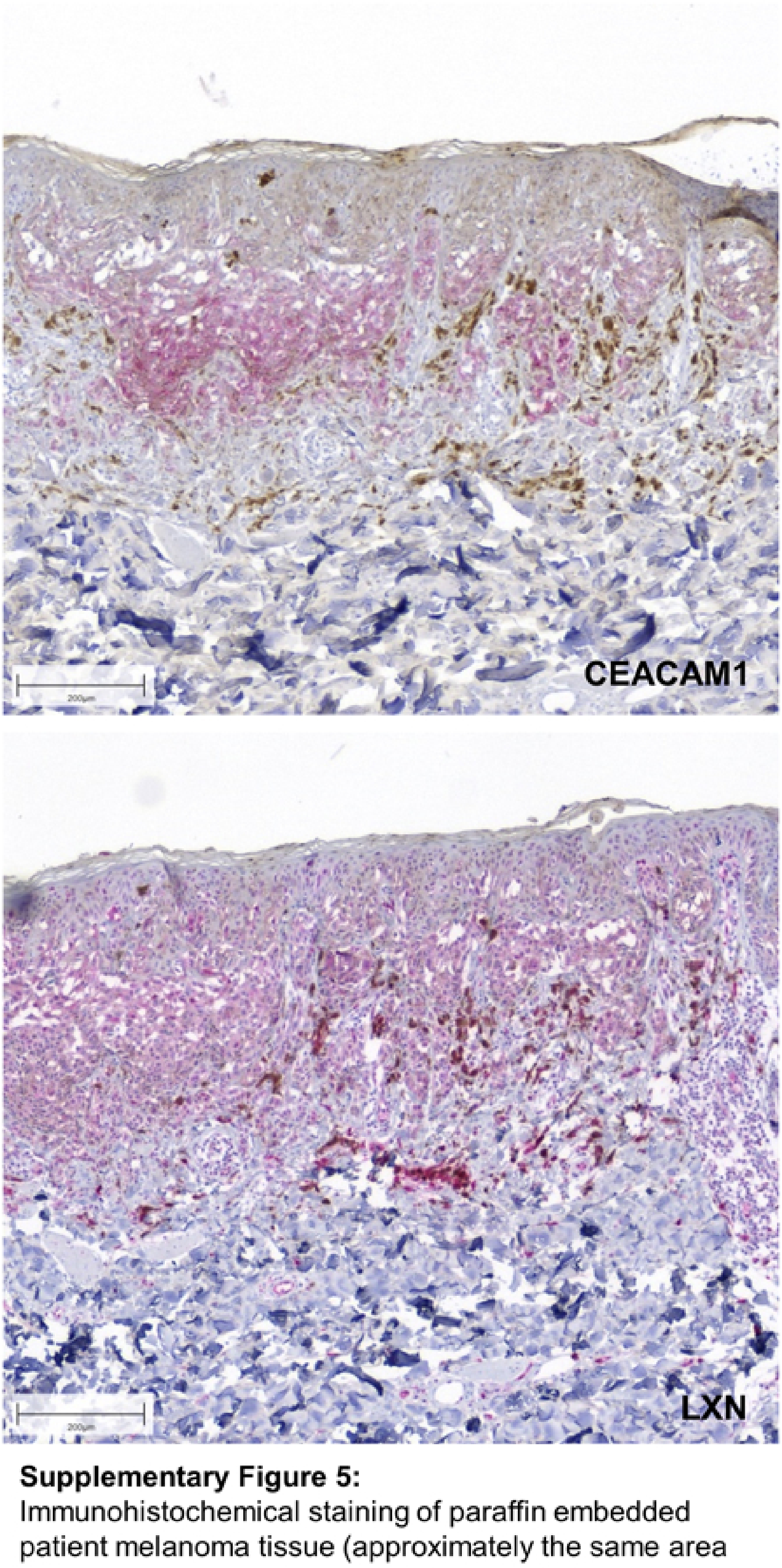

Supplementary Figure 5:
Immunohistochemical staining of paraffin embedded patient melanoma tissue (approximately the same area of the respective melanoma) for CEACAM1 (upper panel) and LXN(lower panel). scale bar: 200µm
Areas with high CEACAM1 and high LXN expression often overlap.

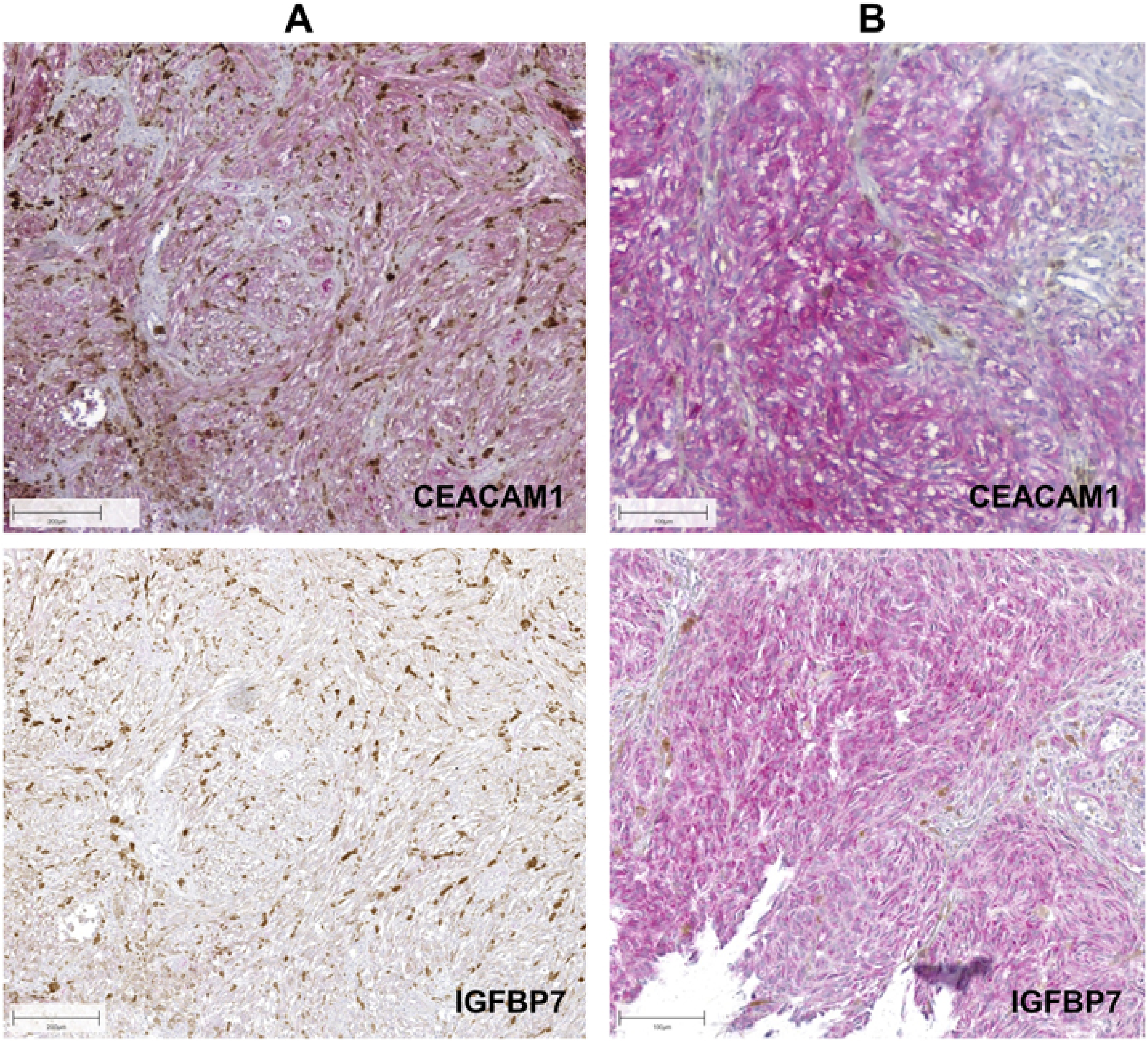

Supplementary Figure 6:
Immunohistochemical staining of two (**A** and **B**) paraffin embedded patient melanoma (approximately the same area of the respective melanoma) for CEACAM1 (upper panels) and IGFBP7 (lower panels). scale bar: 200µm
Areas with high CEACAM1 and high IGFBP7 expression sometimes overlap, but more often do not overlap.

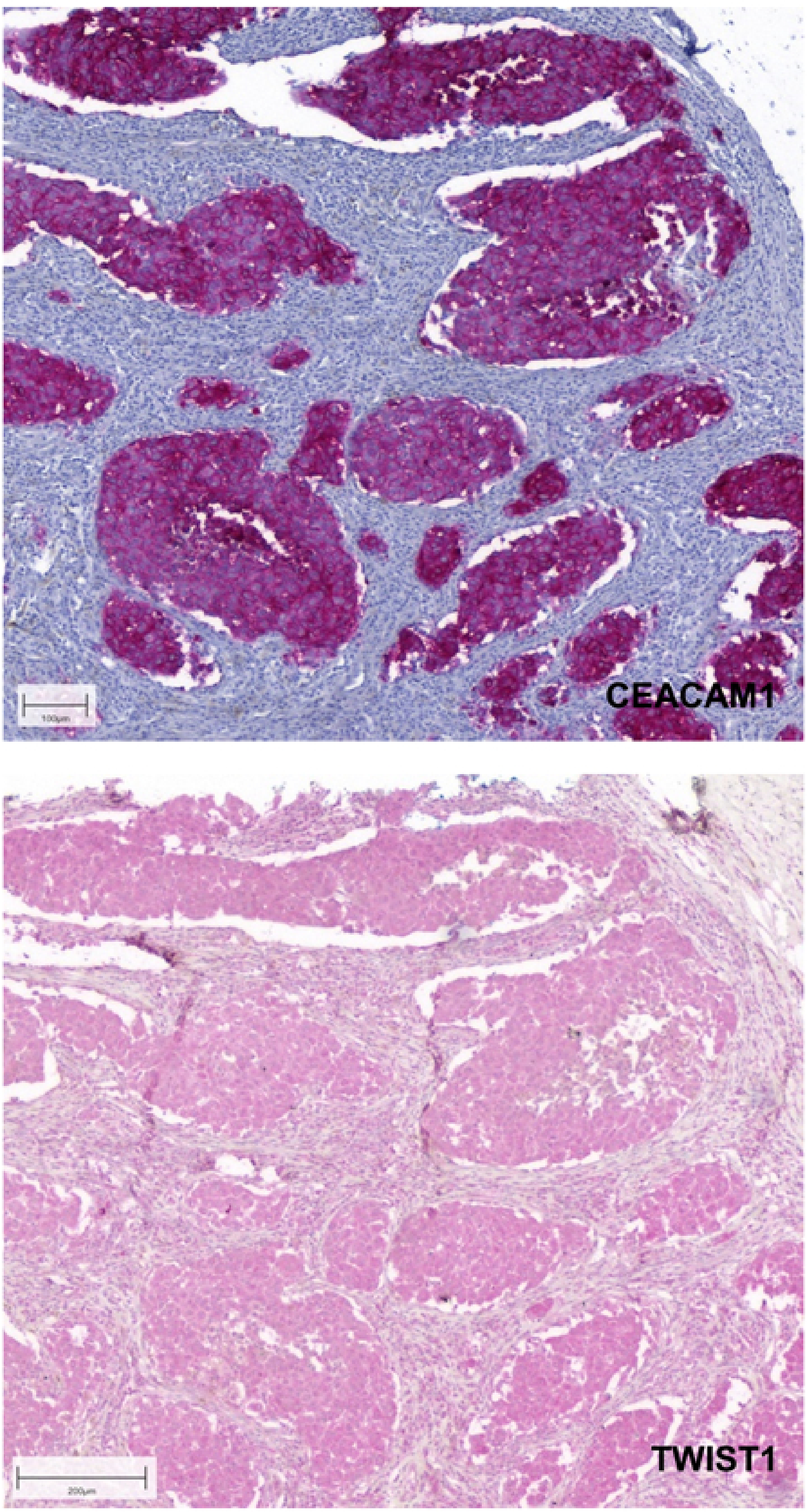

Supplementary Figure 7:
Immunohistochemical staining of paraffin embedded patient melanoma tissue (approximately the same area of the respective melanoma) for CEACAM1 (upper panel) and TWIST1(lower panel). scale bar: 200µm
Areas with high CEACAM1 and high ratios of cytoplasmic/nuclear TWIST1 expression often overlap.

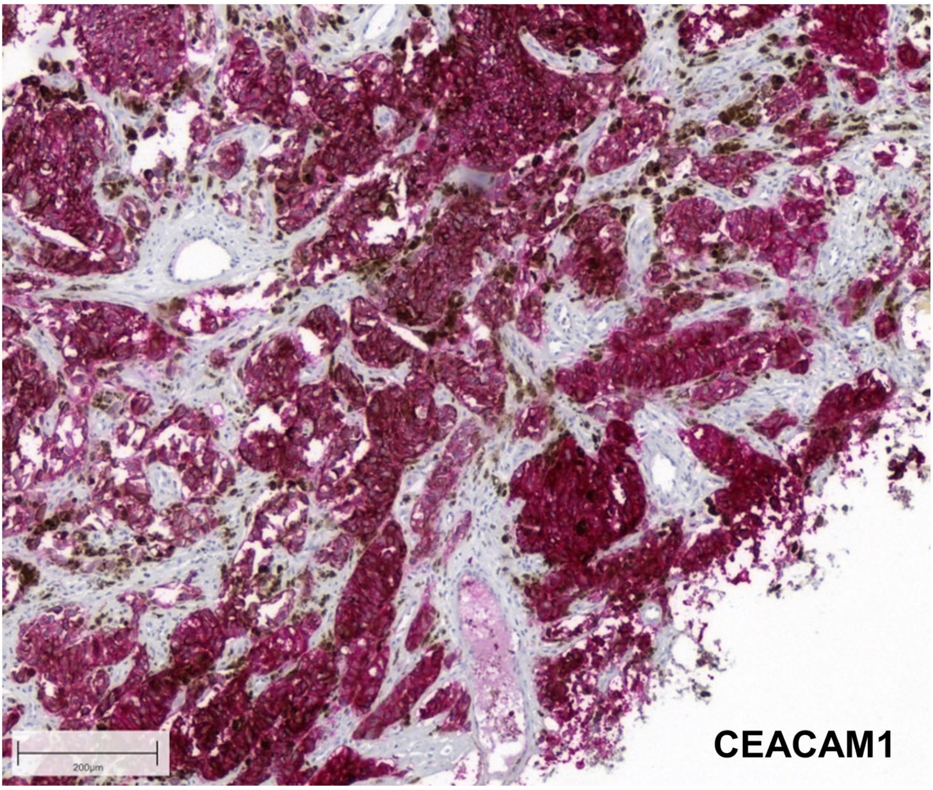

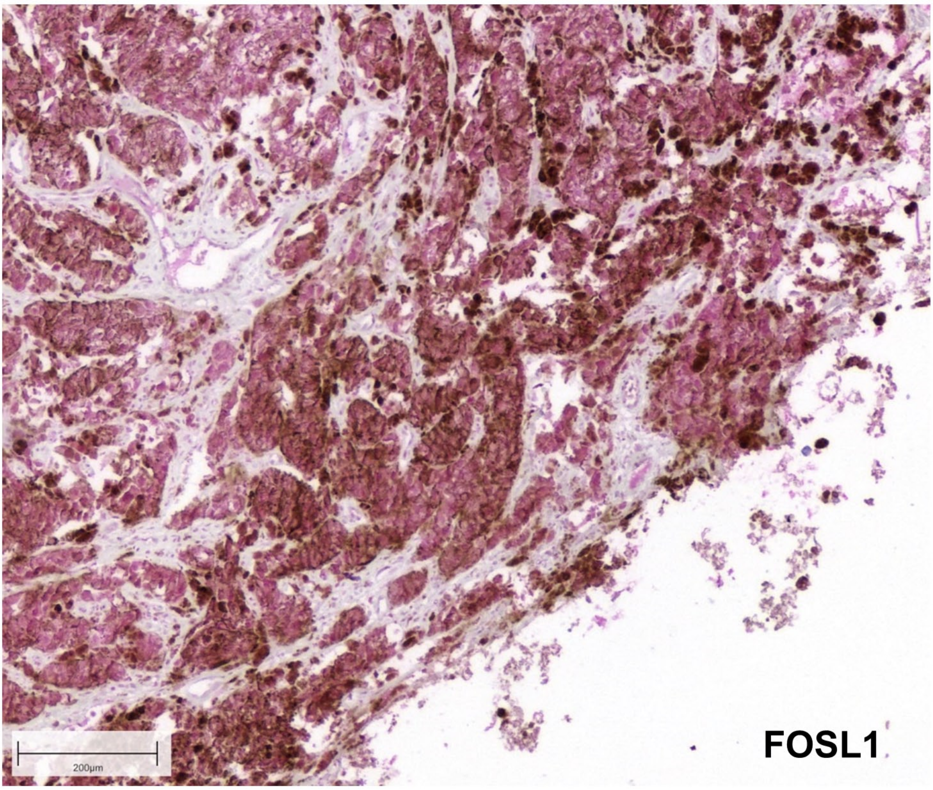

Supplementary Figure 8:
Immunohistochemical staining of paraffin embedded patient melanoma tissue (approximately the same area of the respective melanoma) for CEACAM1 (upper panel) and FOSL1 (lower panel). scale bar: 200µm
Areas with high CEACAM1 and FOSL1 expression often overlap.

Supplementary Table 1: **Comparison of the gene expression of subcutaneous MeWo tumors in scid mice with CEACAM1 knockdown (MeWo CEACAM kd) versus expression of MeWo Luc control (MeWo Luc) tumors. Included are only fold changes at least +/- 1.51; ANOVA and adjusted p-values <0.05.**

Gene:	Fold Change:
LXN	-73.5
KDELC2	-9.2
FKBP11	-8.0
MAGEC1	-7.0
ENPP5	-7.0
ZNF544	-6.1
CYTL1	-6.1
PIBF1	-4.9
C1orf115	-4.9
S100A2	-4.6
LONRF3	-4.6
COL9A3	-4.0
LOC100287628	-4.0
PDLIM4	-3.7
CA2	-3.5
CD58	-3.5
NNMT	-3.5
TNC	-3.2
C8orf48	-3.2
KDELC1	-3.2
EIF1AY	-3.0
TGFBI	-3.0
PLS1	-3.0
ID4	-3.0
IL11	-3.0
LRRIQ3	-3.0
LOC100286909	-2.8
TRIM6	-2.8
AIM2	-2.8
AXL	-2.8
ITGA4	-2.8
SLITRK5	-2.8
SRD5A3	-2.8
HSPA4L	-2.8
SORL1	-2.8
PION	-2.8
HOXA10	-2.6
KLHL13	-2.6

**CYP51A1**	-2.6
**AMIGO2**	-2.6
**GOLGA8A**	-2.6
**SLC22A15**	-2.6
**ZNF567**	-2.6
**SEL1L3**	-2.6
**SNORA61**	-2.6
**ADM**	-2.5
**S100A4**	-2.5
**DKK1**	-2.5
**HSPA6**	-2.5
**CENPE**	-2.5
**ANKRD10**	-2.5
**ARGLU1**	-2.5
**MRAP2**	-2.5
**AHSA2**	-2.5
**UPF3B**	-2.5
**NALCN**	-2.5
**MYOF**	-2.3
**PCDHB15**	-2.3
**STAMBPL1**	-2.3
**ZNF354A**	-2.3
**BCAS2**	-2.3
**GXYLT2**	-2.3
**C9orf64**	-2.3
**AGPAT9**	-2.3
**TMEM158**	-2.3
**C13orf34**	-2.3
**FAM13C**	-2.3
**IL8**	-2.3
**DCUN1D4**	-2.3
**GLRB**	-2.3
**COL16A1**	-2.3
**EVI2A**	-2.3
**CARS2**	-2.3
**SCFD2**	-2.3
**HNRNPA2B1**	-2.3
**PCCA**	-2.1
**COL4A3**	-2.1
**GPR143**	-2.1
**DIAPH3**	-2.1
**KCNN4**	-2.1
**ERCC5**	-2.1
**FAM184A**	-2.1
**C8orf42**	-2.1

**CHN1**	-2.1
**GLT25D2**	-2.1
**AP4B1**	-2.1
**UCHL3**	-2.1
**RAB20**	-2.1
**LYPD1**	-2.1
**COL4A1**	-2.1
**TGDS**	-2.1
**TFPI2**	-2.1
**IL6**	-2.1
**NCRNA00201**	-2.1
**CYR61**	-2.0
**GRB14**	-2.0
**OSBPL3**	-2.0
**ITGA6**	-2.0
**BIVM**	-2.0
**UGGT2**	-2.0
**CEPT1**	-2.0
**CLEC2D**	-2.0
**MMP8**	-2.0
**DEPDC7**	-2.0
**MOSC2**	-2.0
**TCAM1P**	-2.0
**FIP1L1**	-2.0
**SLAIN1**	-2.0
**ATP6V0E2**	-2.0
**PRDX2**	-2.0
**LARP7**	-2.0
**SPRY2**	-2.0
**VEGFA**	-2.0
**LIG4**	-2.0
**NFKBIZ**	-2.0
**NUCB2**	-2.0
**PHTF1**	-2.0
**CEACAM1**	-2.0
**PDGFC**	-2.0
**TPP2**	-2.0
**FAM111B**	-2.0
**TTF2**	-2.0
**TSPAN13**	-2.0
**CXCL1**	-2.0
**COBL**	-2.0
**ATP5F1**	-2.0
**BIRC3**	-2.0
**GOLGA8B**	-1.9

**UPF3A**	-1.9
**DIS3**	-1.9
**PCID2**	-1.9
**TDRD3**	-1.9
**KIF15**	-1.9
**CHL1**	-1.9
**ANXA1**	-1.9
**SGCB**	-1.9
**AHR**	-1.9
**BRCC3**	-1.9
**C17orf60**	-1.9
**LOC152225**	-1.9
**FOXN3**	-1.9
**LOC100131512**	-1.9
**ZNF721**	-1.9
**WDR3**	-1.9
**NPY1R**	-1.9
**USP46**	-1.9
**DRAM2**	-1.9
**HBXIP**	-1.9
**DSP**	-1.9
**FPGT**	-1.9
**ZNF331**	-1.9
**ACYP1**	-1.9
**MATN2**	-1.9
**CHIC2**	-1.9
**PHGDH**	-1.9
**MYO1B**	-1.9
**ARHGEF6**	-1.9
**FOSL1**	-1.7
**TUBGCP3**	-1.7
**PPP1R9A**	-1.7
**SLC16A1**	-1.7
**TMEM165**	-1.7
**IMMP1L**	-1.7
**TIMP3**	-1.7
**CCDC41**	-1.7
**ERRFI1**	-1.7
**NOTCH2**	-1.7
**NDFIP2**	-1.7
**SOX8**	-1.7
**IL12A**	-1.7
**NA**	-1.7
**CCNE2**	-1.7
**MYBL1**	-1.7

**MALAT1**	-1.7
**SEC22B**	-1.7
**ZIC2**	-1.7
**TBC1D4**	-1.7
**LOC643783**	-1.7
**CEP70**	-1.7
**PFKFB4**	-1.7
**C5orf62**	-1.7
**LPXN**	-1.7
**CLK1**	-1.7
**FANCL**	-1.7
**TRIM33**	-1.7
**SNHG12**	-1.7
**HAUS1**	-1.7
**FARP1**	-1.7
**ZNF430**	-1.7
**MYCBP2**	-1.7
**KIAA1644**	-1.7
**PMEPA1**	-1.7
**MTCP1**	-1.7
**MAP7D3**	-1.7
**CNN3**	-1.7
**AHNAK2**	-1.7
**ANKRD6**	-1.7
**MFHAS1**	-1.7
**ELOVL7**	-1.7
**MICALL2**	-1.7
**AKAP12**	-1.7
**ATP1B1**	-1.7
**ATF2**	-1.7
**SCHIP1**	-1.7
**TMOD2**	-1.7
**KRT27**	-1.7
**KCTD12**	-1.7
**PAN2**	-1.7
**NUAK1**	-1.7
**PPAPDC1A**	-1.7
**GPATCH2**	-1.7
**EBP**	-1.7
**ZNF521**	-1.7
**CDC16**	-1.7
**BEX2**	-1.7
**SLAMF7**	-1.6
**SLC16A4**	-1.6
**LAT**	-1.6

CSGALNACT1	-1.6
CEP63	-1.6
FRMD5	-1.6
C4orf32	-1.6
CARKD	-1.6
SEMA3B	-1.6
GKAP1	-1.6
MBNL2	-1.6
RNF182	-1.6
ZDHHC23	-1.6
ARAP2	-1.6
CA8	-1.6
DDX20	-1.6
KIF14	-1.6
WDR77	-1.6
MZT1	-1.6
CDH2	-1.6
LOC730236	-1.6
TSPAN7	-1.6
FHOD3	-1.6
RAP1A	-1.6
ZNF268	-1.6
PIF1	-1.6
MPP6	-1.6
NRAS	-1.6
AADAT	-1.6
SNORD104	-1.6
ID1	-1.6
C13orf27	-1.6
CUL4A	-1.6
ADORA2B	-1.6
GADD45B	-1.6
SPC25	-1.6
CAPZA1	-1.6
MCM9	-1.6
FLJ43663	-1.6
ERO1LB	-1.6
CCNL2	-1.6
HOXA4	-1.6
FBXO25	-1.6
GLIS2	-1.6
RDH11	-1.6
C17orf91	-1.6
EXOC6	-1.6
IER3	-1.6

**SETD7**	-1.6
**ABCC4**	-1.6
**CXCL2**	-1.6
**LAMB1**	-1.6
**FAN1**	-1.6
**KIAA0114**	-1.6
**VDR**	-1.6
**DNAJC3**	-1.6
**FBXL3**	-1.6
**OXCT1**	-1.6
**MITD1**	-1.6
**UBAC2**	-1.6
**LARP1B**	-1.6
**CRIM1**	-1.6
**EFHC1**	-1.6
**ARHGEF10**	-1.6
**DFNB59**	-1.6
**C12orf24**	-1.6
**COL4A2**	-1.6
**CDC14B**	-1.6
**CWF19L2**	-1.6
**EEF1E1**	-1.6
**DOCK11**	-1.6
**ZNF692**	-1.6
**ST8SIA1**	-1.6
**CLN5**	-1.6
**MSRB3**	-1.6
**PHLDA2**	-1.6
**CSDE1**	-1.6
**NUPL2**	-1.6
**TWIST1**	-1.6
**SMC4**	-1.6
**TPM1**	-1.6
**ERI1**	-1.6
**KBTBD3**	-1.6
**RBM26**	-1.6
**ZNF177**	-1.6
**FLJ22536**	-1.6
**HELLS**	-1.6
**PTGR1**	-1.6
**MAP3K4**	-1.6
**CHST2**	-1.5
**LRDD**	-1.5
**ETFDH**	-1.5
**WARS2**	-1.5

**TFDP1**	-1.5
**DLGAP5**	-1.5
**NKAP**	-1.5
**SLC25A37**	-1.5
**DOCK4**	-1.5
**FLNA**	-1.5
**BBS2**	-1.5
**TRMT11**	-1.5
**ATP1A1**	-1.5
**IRAK1BP1**	-1.5
**RSBN1**	-1.5
**C10orf118**	-1.5
**TMCO3**	-1.5
**NUP155**	-1.5
**MRE11A**	-1.5
**RNF219**	-1.5
**CWC27**	-1.5
**SNORA64**	-1.5
**RFC4**	-1.5
**NCAPG**	-1.5
**FAS**	-1.5
**C14orf106**	-1.5
**LAMP2**	-1.5
**SCG2**	-1.5
**PTGFRN**	-1.5
**GDAP2**	-1.5
**KIAA1804**	-1.5
**MIPOL1**	-1.5
**ATP1A1OS**	-1.5
**ZNF697**	-1.5
**CCDC109B**	-1.5
**TBC1D8**	-1.5
**RTKN2**	-1.5
**ZDHHC20**	-1.5
**SDCCAG8**	-1.5
**ADA**	-1.5
**MTHFD2L**	-1.5
**ITGA2**	-1.5
**NMU**	-1.5
**C16orf52**	-1.5
**PPFIA4**	-1.5
**AVL9**	-1.5
**NEDD4**	-1.5
**ANKRD28**	-1.5
**LOC100287482**	-1.5

**STAG1**	-1.5
**RBM34**	-1.5
**HERC4**	-1.5
**CHST6**	-1.5
**SYAP1**	-1.5
**FRMD6**	-1.5
**RPS6KA3**	-1.5
**MAN1A2**	-1.5
**STEAP1**	-1.5
**UHRF2**	-1.5
**GLRX**	-1.5
**EIF2C3**	-1.5
**MTMR9L**	-1.5
**PDE2A**	-1.5
**GPR180**	-1.5
**CLIP4**	-1.5
**SVIL**	-1.5
**SUPT3H**	-1.5
**ZNF789**	-1.5
**SYTL4**	-1.5
**TNFRSF12A**	-1.5
**GLUD2**	-1.5
**TMEM106C**	-1.5
**FAM40B**	-1.5
**MRPS17**	-1.5
**SNX25**	-1.5
**TCIRG1**	-1.5
**PRPF18**	-1.5
**BRAF**	-1.5
**TTC39C**	-1.5
**UBASH3B**	-1.5
**RDH10**	-1.5
**KIAA0895**	-1.5
**DCUN1D2**	-1.5
**ETV1**	-1.5
**RSRC2**	-1.5
**SPAG1**	-1.5
**CUL4B**	-1.5
**OFD1**	-1.5
**COL5A3**	-1.5
**CAMK1**	-1.5
**EPHA2**	-1.5
**FLRT2**	-1.5
**CTTNBP2**	-1.5
**PDGFA**	-1.5

**LRIG3**	-1.5
**KLF12**	-1.5
**CTTN**	-1.5
**KIF16B**	-1.5
**HUS1**	-1.5
**ANLN**	-1.5
**USP53**	-1.5
**UCN2**	-1.5
**RBM41**	-1.5
**METT5D1**	-1.5
**ZNF559**	-1.5
**HMGB3L1**	-1.5
**PRSS23**	-1.5
**CHEK1**	-1.5
**NRIP3**	-1.5
**USP16**	-1.5
**TOX2**	-1.5
**PLA2G16**	-1.5
**POLA1**	-1.5
**DCUN1D1**	-1.5
**FAM40A**	-1.5
**CYFIP2**	-1.5
**DOCK9**	-1.5
**NFE2L3**	-1.5
**CDH11**	-1.5
**C5orf25**	-1.5
**METTL3**	-1.5
**ITGA3**	-1.5
**ABHD3**	-1.5
**COMMD10**	-1.5
**RALGAPA1**	-1.5
**RHOC**	-1.5
**C17orf76**	-1.5
**AGPAT5**	-1.5
**VTI1B**	-1.5
**CXCL3**	-1.5
**TTC26**	-1.5
**THOC1**	-1.5
**SEPT4**	-1.5
**IFI30**	-1.5
**HIPK2**	1.5
**ANXA10**	1.5
**FRMD4B**	1.5
**HOXD1**	1.5
**ZNF440**	1.5

**TTC15**	1.5
**TRDMT1**	1.5
**NFIA**	1.5
**HDX**	1.5
**TNNT1**	1.5
**QPCT**	1.5
**HLA-F**	1.5
**LOC148189**	1.5
**FZD3**	1.5
**CBX4**	1.5
**KLHL29**	1.5
**C14orf93**	1.5
**C22orf39**	1.5
**CCDC113**	1.5
**LOC728769**	1.5
**C21orf91**	1.5
**MTMR6**	1.5
**PXMP4**	1.5
**SEMA3C**	1.5
**PI15**	1.5
**PHF15**	1.5
**NCRNA00094**	1.5
**ITGB1BP3**	1.5
**SEMA6D**	1.5
**LONRF1**	1.5
**FCRLB**	1.5
**PRCC**	1.5
**C6orf192**	1.5
**ANK3**	1.5
**ZNF280B**	1.5
**TP53INP2**	1.5
**C15orf37**	1.5
**ARHGAP31**	1.5
**C1orf198**	1.5
**CCNO**	1.5
**PPP1R3D**	1.5
**IFIT5**	1.5
**C22orf25**	1.5
**ARL5B**	1.5
**CBLB**	1.5
**LPIN1**	1.5
**NAA16**	1.5
**TM4SF4**	1.5
**RFX5**	1.5
**RNASEL**	1.5

ADAR	1.5
FAM53B	1.5
PNRC1	1.5
SOX6	1.5
DCAF7	1.5
TTC30A	1.5
CABLES1	1.5
EXOSC6	1.5
GRAMD4	1.5
HLA-G	1.5
DHRS11	1.5
TIGD2	1.5
CXorf24	1.5
RAP2B	1.5
APOD	1.5
TM4SF18	1.5
ITM2A	1.5
FRAT2	1.5
OTUD1	1.5
FAM167A	1.5
APH1A	1.5
CYBA	1.5
EGLN3	1.5
HPGD	1.5
PGGT1B	1.5
AKAP11	1.5
C1orf85	1.5
TXNIP	1.5
FKBP10	1.5
LOC646762	1.5
PRMT6	1.5
PHLPP1	1.5
TEAD3	1.5
FZD7	1.5
ALG1	1.5
SOCS6	1.5
MORC1	1.5
ISG20L2	1.5
C1S	1.5
SDC2	1.5
TOR3A	1.5
EFHD1	1.5
CDC42EP4	1.5
ZNF512B	1.5
CRISPLD1	1.6

**OCA2**	1.6
**C10orf26**	1.6
**TSSC1**	1.6
**TRIM13**	1.6
**PTGS2**	1.6
**C9orf16**	1.6
**PQLC3**	1.6
**IRX3**	1.6
**RFTN1**	1.6
**ZNF823**	1.6
**ITPKB**	1.6
**BEX4**	1.6
**STON1**	1.6
**ARHGAP15**	1.6
**LGALS3BP**	1.6
**OSTM1**	1.6
**LOC100288092**	1.6
**GLIS1**	1.6
**UGCG**	1.6
**TRAPPC6A**	1.6
**ZNF362**	1.6
**LRCH1**	1.6
**TPPP3**	1.6
**RBM8A**	1.6
**CKAP4**	1.6
**ZNFX1**	1.6
**MIPEP**	1.6
**FTH1**	1.6
**PPIA**	1.6
**NUFIP1**	1.6
**FRAT1**	1.6
**SLFN5**	1.6
**PAIP1**	1.6
**LOC729887**	1.6
**LEPREL4**	1.6
**DCLK1**	1.6
**TRIM63**	1.6
**TSPAN10**	1.6
**PDZRN3**	1.6
**KRTAP7-1**	1.6
**LOC100289219**	1.6
**NCKAP5**	1.6
**NKX2-2**	1.6
**DENND2D**	1.6
**MGC16275**	1.6

**CYBASC3**	1.6
**GMPR**	1.6
**GRN**	1.6
**INPP5A**	1.6
**C13orf31**	1.6
**ZBED3**	1.6
**IRF9**	1.6
**TLR1**	1.6
**HLA-B**	1.6
**CARD6**	1.6
**MR1**	1.6
**TMCC3**	1.6
**C1orf93**	1.6
**ADI1**	1.7
**KCNRG**	1.7
**TTC30B**	1.7
**HEXIM2**	1.7
**ST6GALNAC3**	1.7
**LYZ**	1.7
**STAT5A**	1.7
**STK38L**	1.7
**TLE1**	1.7
**PLP1**	1.7
**LOC255512**	1.7
**HLA-E**	1.7
**MYLIP**	1.7
**FAM53A**	1.7
**ODC1**	1.7
**C16orf54**	1.7
**ACP5**	1.7
**ADAM17**	1.7
**C13orf1**	1.7
**CTNNA2**	1.7
**FABP3**	1.7
**LOC100127888**	1.7
**MLANA**	1.7
**PLXDC2**	1.7
**LRGUK**	1.7
**KIT**	1.7
**NCRNA00052**	1.7
**PCDHB6**	1.7
**CITED1**	1.7
**TMEM140**	1.7
**PPM1K**	1.7
**ZHX2**	1.9

**FXYD5**	1.9
**MAL2**	1.9
**RGS20**	1.9
**PCSK9**	1.9
**PGBD5**	1.9
**UBL3**	1.9
**ELF1**	1.9
**PITX2**	1.9
**KLF11**	1.9
**CREB5**	1.9
**ARHGAP29**	1.9
**C8orf75**	1.9
**DNAJC22**	1.9
**FKBP7**	1.9
**SYK**	1.9
**SPP1**	1.9
**CTSS**	1.9
**PLTP**	1.9
**ID2**	1.9
**CRHBP**	1.9
**FOXO1**	1.9
**RGMB**	1.9
**EPCAM**	1.9
**MGST2**	2.0
**NKAIN2**	2.0
**HOXD4**	2.0
**MYEF2**	2.0
**SNAI2**	2.0
**KBTBD6**	2.0
**APOBEC3G**	2.0
**ACTL8**	2.0
**MX2**	2.0
**PDK4**	2.0
**GABRB3**	2.1
**IFITM2**	2.1
**FAM69C**	2.1
**RASGRP3**	2.1
**CYSLTR2**	2.1
**EOMES**	2.1
**PLCB4**	2.1
**CNDP1**	2.1
**ST7OT1**	2.3
**SERPINF1**	2.3
**DGKI**	2.3
**PDLIM1**	2.3

**FSTL5**	2.3
**STAP2**	2.3
**LOC100288721**	2.3
**SCIN**	2.3
**HAS2**	2.3
**RBPMS2**	2.3
**GDF15**	2.3
**RGS2**	2.5
**C11orf96**	2.5
**INHBA**	2.5
**AGR2**	2.5
**PROCR**	2.5
**APOE**	2.5
**HORMAD1**	2.5
**PLCL1**	2.6
**NEK3**	2.6
**HTN1**	2.6
**QPRT**	2.6
**MPZ**	2.6
**DNAJC15**	2.6
**FBLN5**	2.6
**LPAR6**	2.6
**FAM176A**	2.8
**CPB1**	2.8
**RIPPLY2**	2.8
**TSPAN8**	2.8
**PCDHB2**	3.0
**KBTBD7**	3.0
**HEATR7B1**	3.0
**CD79B**	3.2
**ZNF680**	3.7
**MAGEA12**	4.0
**LUZP4**	4.0
**CTHRC1**	4.3
**CTSK**	4.3
**NCCRP1**	4.6
**PYCARD**	4.9
**FAM133A**	5.3
**VAMP8**	5.3
**IGFBP7**	10.6

Supplementary Table 2: Comparison of the gene expression of subcutaneous MeWo tumors in scid mice with CEACAM1 knockdown (MeWo CEACAM kd) versus expression of MeWo Luc control (MeWo Luc) tumors with mean Signal strengths of the respective groups and Affymetrix Probe Set IDs.

Probe Set ID	GENE_SYMBOL	Signal mean - MeWo Luc (Baseline)	Signal mean - MeWo CEACAM kd	Raw p value	Adj p value	Fold Change
214907_at	CEACAM21	4.008149	3.9612358	0.707944	0.707944	-1.0330522
216605_s_at	CEACAM21	4.299334	4.456324	0.2854962	0.2854962	1.11495849
239317_at	CEACAM21	3.0174503	2.9318337	0.32589346	0.32589346	-1.0611412
208052_x_at	CEACAM3	6.1682215	6.245122	0.0834202	0.0834202	1.05474957
210789_x_at	CEACAM3	6.0557785	5.9334345	0.18361485	0.18361485	-1.0885019
201884_at	CEACAM5	4.358782	4.356951	0.9902916	0.9902916	-1.0012699
217291_at	CEACAM5	5.9122486	5.8941493	0.8218636	0.8218636	-1.0126245
203757_s_at	CEACAM6	3.7025256	3.8152652	0.3391133	0.3391133	1.08127958
211657_at	CEACAM6	4.875628	5.1295133	0.10071793	0.10071793	1.19241407
206676_at	CEACAM8	3.1470895	3.0945938	0.4906266	0.4906266	-1.0370574
